# Potential Molecular Mechanisms of Ginsenosides for Alleviating Cancer-Related Fatigue and Clinical Research Progress of Ginseng Preparations: A Review

**DOI:** 10.3390/molecules31213917

**Published:** 2026-10-30

**Authors:** Jiaying Guo, Qin Feng, Kunshuang Shen, Jie Cheng, Yuying Zhu, Yinling Luan, Jingzhe Gao, Shengnan Zhu, Fuxiang Huang, Ning Zhang, Zhigang Wang, Hui Sun

**Affiliations:** 1 Metabolomics Laboratory, Department of Pharmaceutical Analysis, College of Pharmacy, National TCM Key Laboratory of Serum Pharmacochemistry, National Chinmedomics Research Center, State Key Laboratory of Integration and Innovation of Classic Formula and Modern Chinese Medicine, Heilongjiang University of Chinese Medicine, 24 Heping Road, Harbin 150040, China; mohwanko2002@163.com (J.G.); 18800462824@163.com (K.S.); luan00987@163.com (Y.L.); 15075560069@163.com (J.G.); edwardzhu370@outlook.com (S.Z.); hfx3194457279@163.com (F.H.); 2 Shandong Key Laboratory of Gelatine Medicines Research and Development, Dong’e Ejiao Co., Ltd., Ejiao Road 78, Dong’e, Liaocheng 252200, China; fengqin0124@163.com (Q.F.); chengjie@dongeejiao.com (J.C.); zhuyuying23@dongeejiao.com (Y.Z.)

**Keywords:** cancer-related fatigue (CRF), Panax ginseng, ginsenoside Rg1, ginsenoside Rg3, ginsenoside Rb1, inflammation, molecular mechanisms, clinical studies

## Abstract

Cancer-related fatigue (CRF) is a persistent, subjective symptom experienced by patients with cancer over the course of the disease and its treatment. It can substantially impair patients’ quality of life, and its pathophysiological mechanisms remain incompletely understood. Ginseng is a traditional Chinese medicinal herb used to tonify qi and alleviate debility associated with prolonged illness. Clinical studies suggest that ginseng preparations may alleviate CRF to some extent. However, substantial heterogeneity among existing studies means that robust and reproducible evidence supporting the efficacy of ginseng in alleviating CRF remains insufficient. Ginsenosides are the major bioactive constituents of ginseng. Studies have reported several pharmacological effects of ginsenosides relevant to CRF, including anti-inflammatory effects and modulation of neuroendocrine function and muscle energy metabolism. These findings suggest that ginsenosides may have the potential to alleviate CRF. This review focuses on three representative ginsenosides (Rg1, Rg3, and Rb1), explores their potential molecular mechanisms in CRF alleviation, and reviews clinical studies of ginseng preparations for CRF management. Based on gaps in the current evidence, this review identifies priorities for future research to inform further studies of ginseng-based interventions for CRF.

## 1. Introduction

Cancer comprises a group of highly lethal malignancies with a high global prevalence. Cancer-related fatigue (CRF) is one of the most common symptoms experienced by patients with cancer. The National Comprehensive Cancer Network defines CRF as persistent subjective fatigue affecting the physical, emotional, or cognitive domains and attributable to cancer itself or its treatment [1]. Available evidence indicates that the overall prevalence of CRF is 60% or higher [2], reaching 80–99% among patients receiving radiotherapy or chemotherapy [3]. Unlike ordinary fatigue, CRF is not relieved by rest and is not clearly associated with recent activity levels [4]. It may also persist for months or even years after the completion of cancer treatment [5], substantially impairing patients’ quality of life and ability to perform daily activities [6]. Current interventions for CRF can be broadly classified into pharmacological and non-pharmacological approaches. Non-pharmacological interventions include exercise [7,8,9,10,11,12,13,14,15,16], psychological interventions [17,18,19,20,21], complementary and alternative therapies [22,23,24,25,26], and nutritional supplementation [27,28,29]. Pharmacological treatments include psychostimulants, corticosteroids, erythropoietin, and antidepressants [30,31,32,33,34,35,36,37,38,39]. Although these interventions may alleviate CRF to some extent, both approaches have limitations. Some non-pharmacological interventions require active patient participation and sustained adherence, whereas pharmacological treatments may cause adverse effects. Therefore, identifying potential therapeutic agents with consistent efficacy and a favorable safety profile remains an important objective in CRF research.

Traditional Chinese medicine (TCM) has a long history and is characterized by a distinctive theoretical framework and an approach to diagnosis based on pattern differentiation [40]. In recent years, TCM has attracted increasing attention for its potential role in managing CRF. Ginseng is one of the herbal medicines commonly used in clinical practice to alleviate CRF [41,42,43]. It has long been used in Asian countries, including China, South Korea, and Japan, owing to its notable pharmacological effects [44,45]. Research has identified ginsenosides as the primary bioactive constituents responsible for the pharmacological activities of ginseng [46]. Ginsenosides are triterpenoid saponins classified into dammarane and oleanane types according to their aglycone skeletons. Dammarane-type ginsenosides are further divided into three subtypes: protopanaxadiol, protopanaxatriol (PPT), and ocotillol [47,48]. Among species of the genus Panax, Asian ginseng (*Panax ginseng* C. A. Mey.) and American ginseng (*Panax quinquefolius* L.) are the most widely used and extensively studied. The two species differ in total ginsenoside content and in the composition of their major ginsenosides [49]. Asian ginseng can be processed into sun-dried ginseng by direct drying or into red ginseng and black ginseng through steaming processes. Korean red ginseng refers specifically to red ginseng produced in Korea. Steaming induces structural transformations in ginsenosides, resulting in a markedly different ginsenoside profile from that of sun-dried ginseng. These transformations constitute the material basis for the distinctive pharmacological effects of processed ginseng [50]. The ginsenoside profiles of different Panax species and their processed products are summarized in Table 1 [51,52,53].

In recent years, numerous studies have reported that ginsenosides exhibit a range of biological activities, including neuroprotective, anticancer, antioxidant, immunomodulatory, anti-inflammatory, and antifatigue effects [54,55,56,57,58]. However, direct evidence regarding the effects of ginsenosides on CRF remains limited. Clinical studies investigating interventions for CRF have predominantly examined ginseng preparations rather than individual ginsenosides. Consequently, a gap remains between the potential mechanisms of action of ginsenosides and the clinical findings from studies of ginseng preparations. The scope and focus of the existing literature also warrant further synthesis, as few reviews have integrated evidence on the pathophysiology of CRF, the potential mechanisms of ginsenosides, and the clinical findings for ginseng preparations. Accordingly, this narrative review outlines the major pathophysiological processes implicated in CRF and examines the potential molecular mechanisms of three representative ginsenosides: Rg1, Rb1, and Rg3. It also reviews clinical studies of ginseng preparations for CRF and evaluates the characteristics and limitations of the available evidence. Particular attention is given to the relationship between preclinical findings and clinical evidence, the standardization of ginseng preparations, and the assessment of treatment efficacy. This review aims to inform future research on ginseng and its bioactive constituents as potential interventions for CRF.

## 2. Pathogenesis of Cancer-Related Fatigue (CRF)

**Figure 1:**
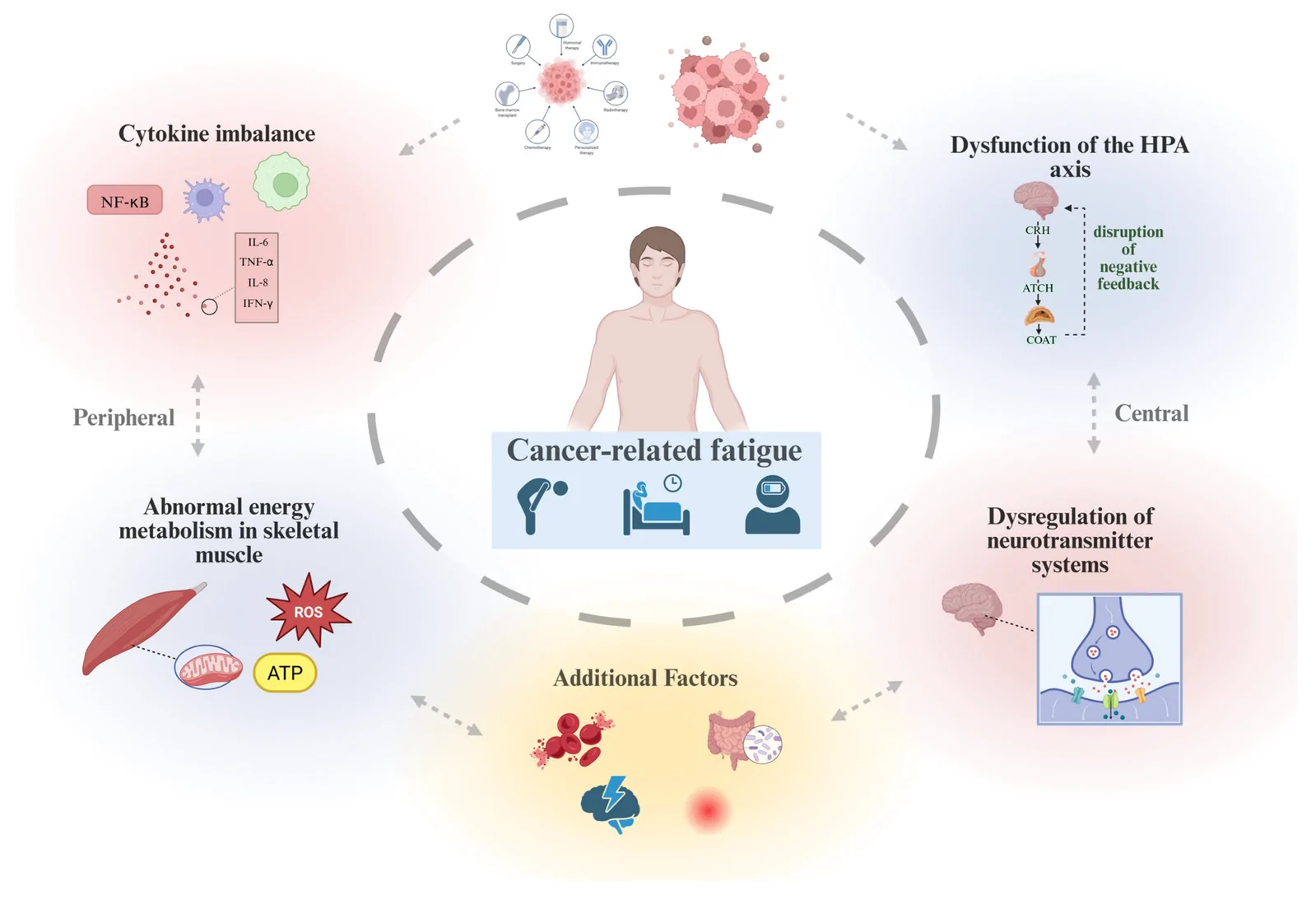
Current hypotheses regarding the pathogenesis of cancer-related fatigue (CRF) implicate imbalances in inflammatory mediators, dysfunction of the hypothalamic–pituitary–adrenal (HPA) axis, dysregulation of neurotransmitter signaling, and impaired ATP metabolism in skeletal muscle. Depression, anemia, and gut microbiota dysbiosis may also be associated with CRF. These mechanisms and associated factors may interact with one another and collectively contribute to the characteristic manifestations of CRF, including persistent fatigue, cognitive impairment, sleep disturbances, and reduced physical functioning. Bidirectional dashed arrows indicate potential interactions, whereas unidirectional dashed arrows indicate a potential mediating role. Created in BioRender.

The pathogenesis of CRF is complex and multifactorial, involving cancer itself, cancer treatments, and psychological factors such as anxiety and depression [42]. Rather than arising from a single pathway, CRF involves a multifactorial pathological process that engages both central and peripheral mechanisms. These include inflammatory responses, dysfunction of the hypothalamic–pituitary–adrenal (HPA) axis, imbalances in central neurotransmitters, impaired mitochondrial energy metabolism, and reduced adenosine triphosphate (ATP) synthesis in skeletal muscle. These mechanisms interact through complex regulatory networks and collectively contribute to the development and progression of CRF [59,60,61,62]. Imbalances in inflammatory mediators may contribute to HPA axis dysfunction and skeletal muscle abnormalities, thereby promoting CRF through both central and peripheral mechanisms (Figure 1); the evidence sources discussed in this section are classified and summarized in Table 2.

### 2.1. Inflammatory Cytokine Imbalance

Dysregulation of inflammatory cytokines is considered one of the major hypotheses underlying CRF pathogenesis. Cancer itself and anticancer treatments can cause tissue damage, thereby triggering sustained immune activation and chronic inflammatory responses. These processes may lead to abnormal levels of inflammatory mediators and markers, including the pro-inflammatory cytokines tumor necrosis factor-α (TNF-α), interleukin-6 (IL-6), and interleukin-8, as well as C-reactive protein [63,64,65,66]. Clinical studies have also shown that the levels of certain inflammatory markers are associated with CRF severity (H-CRF) [67,68], suggesting that inflammatory responses may contribute to the development of fatigue.

Current evidence suggests that dysregulated inflammatory signaling may contribute to CRF through multiple pathways. The canonical nuclear factor-κB (NF-κB) signaling pathway is widely implicated in inflammatory processes. In response to inflammatory stimuli, activation of IκB kinase (IKK) promotes the degradation of inhibitor of κB (IκB), thereby facilitating NF-κB translocation into the nucleus and the transcription of pro-inflammatory genes [69,70]. In some inflammatory diseases and cancer-related models, NF-κB activation has been associated with increased expression of pro-interleukin-1β and NOD-, LRR- and pyrin domain-containing protein 3 (NLRP3). Elevated levels of reactive oxygen species (ROS) and other danger signals can promote inflammasome assembly, the maturation and release of interleukin-1β (IL-1β), and the subsequent amplification of inflammatory responses [71,72]. Fatigue is a common clinical manifestation of several conditions, including cancer, chronic fatigue syndrome, cachexia, and rheumatoid arthritis. Fatigue in different disease contexts may involve partially overlapping inflammatory and immune-regulatory mechanisms [66,73,74]. Clinical transcriptomic studies have reported increased NF-κB-related transcriptional activity in the peripheral blood of patients with CRF, accompanied by suppression of anti-inflammatory pathways (H-CRF) [75,76]. In addition, a previous study found that metabotropic glutamate receptor 5 signaling in T cells can enhance radiotherapy-induced NF-κB transcriptional activation, suggesting a potential link between this signaling pathway and treatment-related CRF (H-CRF) [77]. Collectively, these findings suggest that NF-κB may represent an important molecular feature of inflammatory responses in CRF. Beyond NF-κB signaling, IL-6-related pathways may also contribute to the inflammatory processes associated with CRF. Upon binding to its receptor, IL-6 activates the Janus kinase (JAK)/signal transducer and activator of transcription 3 (STAT3) pathway. Suppressor of cytokine signaling 3 provides negative feedback to help maintain inflammatory homeostasis [78]. In cancer cachexia, this regulatory balance may be disrupted, resulting in sustained STAT3 activation and chronic inflammation [79,80]. Cachexia and CRF share some pathological features. Moreover, a meta-analysis of inflammatory biomarkers in CRF found a significant positive association between CRF severity and peripheral IL-6 levels [81]. These findings suggest that dysregulation of the IL-6/JAK/STAT3 pathway may represent an important link between inflammatory responses and CRF. Given the involvement of STAT3 signaling in both the central nervous system and skeletal muscle, inflammatory responses may also contribute to disturbances in central regulation and energy metabolism.

Inflammatory dysregulation may also involve alterations in immune cell function. Chronic inflammation and oxidative stress within the tumor microenvironment may promote the polarization of monocytes toward a pro-inflammatory M1-like phenotype, increasing the production of TNF-α, IL-1β, and ROS. These conditions may also impair the reparative functions of M2 macrophages, thereby disrupting inflammatory homeostasis [82]. However, recent evidence suggests that monocytes are not the sole contributors to inflammatory regulation in CRF. A longitudinal transcriptomic study in patients with breast cancer found that type I interferon signaling was associated with the trajectory of CRF development, whereas NF-κB-related inflammatory changes were influenced by peripheral monocyte abundance and became more pronounced during the late post-treatment period (H-CRF) [83]. Furthermore, an animal study demonstrated that Toll-like receptor 4 (TLR4) in skeletal muscle cells can directly recognize tumor-derived damage-associated molecular patterns, thereby inducing chronic inflammation independently of changes in monocyte abundance (C-IF) [84]. Collectively, these findings suggest that monocytes may contribute to CRF pathogenesis but are unlikely to be the sole determinants of inflammatory dysregulation. They also highlight potential noncanonical inflammatory pathways that may be involved in CRF.

Peripheral inflammatory signals may also affect central nervous system (CNS) function through several pathways, including alterations in blood–brain barrier (BBB) integrity, microglial activation, and stimulation of vagal pathways. These effects may contribute to neurotransmitter imbalances and impaired energy metabolism, potentially leading to symptoms such as fatigue, sleep disturbances, and pain [85,86]. Clinical studies have shown that the systemic immune-inflammation index is positively associated with CRF severity [87]. Therefore, inflammatory dysregulation may serve as an important upstream mechanism linking cancer-associated immune activation with CNS dysfunction and peripheral metabolic disturbances.

### 2.2. Dysregulation of the Hypothalamic–Pituitary–Adrenal (HPA) Axis

The HPA axis is a key neuroendocrine system involved in regulating stress responses and glucocorticoid homeostasis. It comprises the hypothalamus, pituitary gland, and adrenal glands [88]. Current hypotheses suggest that HPA axis dysregulation may contribute to the development and progression of CRF through alterations in cortisol rhythms and impaired glucocorticoid receptor (GR) function [89,90]. However, alterations in HPA axis function do not follow a uniform pattern and may be influenced by several factors, including inflammation, disease type, and persistent stress.

Cortisol, plays essential roles in inflammatory regulation, energy metabolism, and stress responses [59], which is the primary glucocorticoid hormone among the human beings. With its levels regulated through negative feedback mechanisms [91], cortisol exhibits a stable circadian rhythm, under the physiological conditions. Clinical studies have shown that patients with breast cancer who experience CRF exhibit elevated nocturnal cortisol levels and flattened diurnal cortisol rhythms. These associations are not fully explained by depressive symptoms. Moreover, patients undergoing active treatment may exhibit elevated cortisol levels throughout the day, whereas those with persistent fatigue after treatment completion primarily exhibit disrupted cortisol rhythms. These findings suggest that alterations in HPA axis rhythmicity may contribute to the persistence of CRF (H-CRF) [92,93]. In addition to abnormal cortisol secretion, alterations in GR function may also contribute to HPA axis dysregulation. Chronic inflammation may reduce GR sensitivity, thereby weakening glucocorticoid-mediated anti-inflammatory regulation and further disrupting inflammatory homeostasis and stress responses [94,95,96]. A clinical study in patients with ovarian cancer found that reductions in IL-6 and nocturnal cortisol levels after treatment coincided with improvements in CRF, depressive symptoms, and physical functioning. These findings suggest that inflammatory responses and HPA axis dysregulation may interact bidirectionally in the context of CRF (H-CRF) [97].

Animal studies suggest that alterations in the HPA axis associated with CRF are highly context-dependent. In lipopolysaccharide (LPS)-induced inflammatory and 4T1 breast cancer models, reduced hypothalamic corticotropin-releasing hormone (CRH) peptide expression has been observed alongside decreases in serum CRH and cortisol levels. These findings suggest that chronic inflammation or tumor burden may lead to adaptive suppression of the HPA axis (C-IF) [98]. In contrast, cisplatin-induced chemotherapy models have shown increased CRH expression and elevated cortisol or corticosterone levels, suggesting that acute treatment-related stress may trigger excessive HPA axis activation (CH-IF) [99]. Thus, HPA axis dysregulation may not represent a fixed pathological state but rather a dynamic process that varies with disease progression and treatment-related stimuli. HPA axis dysfunction may affect inflammatory responses, sleep rhythms, and energy metabolism. It may also interact with the 5-hydroxytryptamine (5-HT) system, jointly influencing fatigue perception and functional status [100]. Taken together, these findings suggest that HPA axis dysregulation may represent an important neuroendocrine mechanism underlying CRF. However, the heterogeneous patterns of HPA axis alterations highlight the need for further research to clarify the relationship between HPA axis dysregulation and CRF across different disease stages and treatment contexts.

### 2.3. Neurotransmitter Dysregulation

Alterations in central neurotransmitter homeostasis may contribute to the development and progression of CRF. Monoamine neurotransmitters in the brain, including 5-HT, dopamine (DA), and noradrenaline (NE), may influence fatigue perception and behavioral activity [101,102,103].

The 5-HT system is considered an important candidate for the neural regulation of fatigue among these neurotransmitter systems. Several pathways may contribute to 5-HT system dysregulation during cancer progression and treatment. For example, inflammation can activate indoleamine 2,3-dioxygenase (IDO), promoting the diversion of tryptophan (TRP) metabolism toward the kynurenine pathway. This reduces the availability of TRP for 5-HT synthesis and may alter central 5-HT signaling [60,90,103,104]. In addition, elevated cortisol levels associated with HPA axis dysregulation may activate tryptophan 2,3-dioxygenase, further shifting TRP metabolism and altering brain 5-HT levels [105]. Increased peripheral TRP levels may also enhance TRP transport across the BBB, potentially contributing to changes in central 5-HT levels [106]. These mechanisms suggest that changes in central 5-HT levels may vary depending on the underlying physiological and pathological context. Animal studies have shown that TRP accumulation in the brain can selectively increase 5-HT levels in the preoptic area and promote central fatigue during physical activity (NC-F) [107,108]. Studies of myalgic encephalomyelitis/chronic fatigue syndrome have also suggested that excessive central 5-HT activity may coexist with HPA axis abnormalities, providing insights into the neuroimmune mechanisms potentially associated with fatigue (NC-F) [109]. However, direct measurement of neurotransmitter levels at synapses in the human brain remains challenging, and findings regarding central 5-HT levels are inconsistent across studies. Therefore, the current evidence supporting the 5-HT hypothesis in CRF remains insufficient.

Other neurotransmitters may also contribute to the regulation of CRF-related symptoms, beyond the 5-HT system. DA plays important roles in motor control, motivation, and reward-related behaviors [110]. Inflammatory mediators may reduce central DA synthesis and synaptic availability through several mechanisms, including depletion of tetrahydrobiopterin, a cofactor required for DA synthesis; upregulation of the dopamine transporter; and activation of the IDO pathway [111,112]. In a mouse model of cachexia, IL-6 was shown to suppress DA release in the mesolimbic pathway, potentially contributing to fatigue (CC) [113]. These findings suggest that interactions between inflammation and neurotransmitter signaling may be involved in CRF pathogenesis. NE, an important neurotransmitter in the sympathetic nervous system, is involved in stress responses and energy metabolism. Cancer-related stimuli may trigger excessive NE release, which can activate β-adrenergic receptors in skeletal muscle and adipose tissue, potentially promoting muscle wasting. Furthermore, elevated NE levels have been reported in individuals experiencing fatigue, suggesting that excessive noradrenergic signaling may contribute to fatigue [114,115]. A study of breast cancer survivors found that patients with CRF exhibited autonomic dysfunction and elevated NE levels associated with sympathetic activity, suggesting that impaired neuroendocrine regulation may contribute to CRF pathogenesis (H-CRF) [116]. In general, central neurotransmitter dysregulation may contribute to CRF by affecting emotional regulation, sleep patterns, motivation for physical activity, and fatigue perception. It may also interact with inflammatory responses and HPA axis dysregulation, forming an interconnected regulatory network.

### 2.4. Mitochondrial Dysfunction and Skeletal Muscle Dysfunction

In addition to immune and inflammatory processes and central nervous system dysfunction, mitochondrial and skeletal muscle dysfunction have attracted considerable attention as potential peripheral mechanisms underlying CRF. Both may contribute to the development of fatigue. Mitochondria are the primary sites of ATP production in skeletal muscle. Inflammatory stimuli, oxidative stress, and other factors may induce mitochondrial abnormalities, including reductions in mitochondrial DNA (mtDNA) levels. These alterations may impair ATP synthesis and compromise normal muscle function [117,118,119].

Current evidence suggests that the cancer-associated inflammatory environment may contribute to CRF development by altering mitochondrial metabolic networks. In cachexia models, inflammatory mediators can upregulate phosphodiesterase 4 expression in skeletal muscle. This upregulation may suppress the cyclic adenosine monophosphate (cAMP)/protein kinase A (PKA)/cAMP response element-binding protein 1 (CREB1) signaling pathway, thereby impairing the regulation of mitochondrial function. These changes may lead to reduced mtDNA levels, respiratory dysfunction, and decreased ATP production (CC, IV) [120]. Anticancer treatments may also disrupt mitochondrial homeostasis. Clinical studies have reported reduced mtDNA copy numbers in the peripheral blood of patients receiving chemotherapy, and this reduction has been associated with the physical dimension of CRF. Radiotherapy may damage mitochondrial complex III in peripheral monocytes, suppress oxidative phosphorylation (OXPHOS), and reduce ATP production. When radiotherapy is combined with androgen deprivation therapy, central mitochondrial abnormalities and anemia may further exacerbate symptoms (H-CRF) [121,122,123]. Animal studies have shown that doxorubicin induces time-dependent changes in skeletal muscle mitochondrial function. Functional impairment, rather than a reduction in mitochondrial abundance, appears to be the predominant finding. In vitro studies have also suggested that doxorubicin may directly impair muscle contractility by disrupting calcium homeostasis in the sarcoplasmic reticulum [124,125]. However, the mitochondrial status of human skeletal muscle tissue cannot be directly extrapolated from findings obtained in animal and in vitro studies, or from mitochondrial measurements in peripheral blood. In addition to impaired energy supply, dysregulated skeletal muscle protein metabolism may be an important component of the peripheral mechanisms underlying CRF. The ubiquitin–proteasome system (UPS) is a major pathway responsible for muscle protein degradation. Tumor-associated inflammatory cytokines and soluble mediators, including proteolysis-inducing factor (PIF), may activate the UPS via multiple mechanisms. Inflammatory cytokines suppress Akt signaling, thereby releasing forkhead box O (FoxO) proteins from Akt-dependent inhibition, and activate STAT3 and other catabolic signaling pathways. These changes may promote the expression of muscle-specific E3 ubiquitin ligases, including Atrogin-1 and muscle RING finger 1 (MuRF1). PIF may directly increase the expression of ubiquitin, E2 enzymes, and 26S proteasome components, thereby accelerating the degradation of contractile proteins. Collectively, these processes may contribute to muscle atrophy and exacerbate fatigue (CC, IV) [126,127]. In addition to the UPS, the calcium-dependent calpain system and the autophagy–lysosome pathway also contribute to muscle protein degradation. Tumor burden may activate calpains and reduce calpastatin abundance. It may also induce excessive autophagy in skeletal muscle while impairing mitochondrial clearance through mitophagy. These processes may interact with the UPS to form a coordinated regulatory network that promotes muscle protein degradation (CC) [128,129,130]. Animal studies have shown that cisplatin can disrupt skeletal muscle homeostasis independently of tumor burden. Potential mechanisms include UPS activation, dysregulated autophagy, disruption of calcium homeostasis, oxidative stress, and mitochondrial damage. These changes may contribute to skeletal muscle atrophy and physical fatigue. However, further clinical research is needed to determine the extent to which these preclinical findings apply to patients with CRF [131]. Overall, mitochondrial dysfunction and skeletal muscle metabolic abnormalities may be among the key peripheral links between tumor burden, treatment-related injury, and physical fatigue.

### 2.5. Additional Contributing Factors

In addition to the potential mechanisms described above, several other factors may contribute to the onset and persistence of CRF symptoms. These include gut microbiota dysbiosis (H-CRF) [132,133,134], anemia (H-CRF) [135], thyroid dysfunction (H-CRF) [136], disease-related pain (H-CRF) [137], and stress, anxiety, and depressive symptoms experienced by patients during cancer diagnosis and treatment (H-CRF) [138,139]. Overall, CRF is a complex clinical phenotype driven by interactions among multiple systems. It is often regarded as the second most common symptom after pain among patients with cancer. However, the specific causal relationships underlying CRF remain to be clarified through further research.

## 3. Potential Molecular Mechanisms by Which Ginsenosides Alleviate CRF

There remains a need to identify therapeutic agents and bioactive compounds that may help alleviate CRF. Current studies suggest that ginsenosides exhibit anti-inflammatory effects, modulate neuroendocrine function, improve energy metabolism, and exert protective effects on hematopoietic function. The molecular targets and signaling pathways identified in different experimental models may provide mechanistic insights into the potential of ginsenosides to alleviate CRF symptoms. Accordingly, this section reviews the available evidence regarding the potential molecular mechanisms through which representative ginsenosides, particularly Rg1, Rg3, and Rb1, may influence pathological processes associated with CRF. The evidence from studies investigating the potential of ginsenosides to alleviate CRF is classified and summarized in Table 3.

### 3.1. Anti-Inflammatory Effects

Current evidence suggests that the anti-inflammatory mechanisms of ginsenosides may primarily involve the TLR4/NF-κB and MAPK signaling pathways, as well as the NLRP3 inflammasome [140,141]. By modulating the release of pro-inflammatory mediators and the functions of immune cells, ginsenosides may help regulate both peripheral and central inflammatory responses. In a mouse model of CRF, the fermented ginseng extract BST204, administered at doses of 100 and 200 mg/kg, reduced the levels of pro-inflammatory cytokines, including TNF-α and IL-6, in peripheral blood and macrophages of chemotherapy-treated mice. BST204 also alleviated chemotherapy-related fatigue and systemic injury (C-M) [142]. Given that BST204 is rich in the ginsenosides Rh2 and Rg3, these findings suggest that these constituents may contribute to the modulation of inflammatory responses associated with CRF. Ginsenosides have demonstrated substantial anti-inflammatory effects in other inflammatory models, providing mechanistic insights into their potential to alleviate CRF.

#### 3.1.1. Potential Anti-Inflammatory Mechanisms of Ginsenoside Rg1

Ginsenoside Rg1 may suppress the TLR4/NF-κB/NLRP3 signaling axis, reducing IL-1β and interleukin-18 release and alleviating LPS-induced septic myocardial injury in mice (NC-M) [143]. In a mouse model of 2,4,6-trinitrobenzenesulfonic acid-induced colitis, Rg1 and the gut microbiota-derived metabolites Rh1 and 20(S)-PPT (20 mg/kg) reduced colonic NF-κB, inducible nitric oxide synthase (iNOS) and cyclooxygenase-2 expression, suppressed pro-inflammatory cytokine release, and modulated the Th17/Treg balance. In vitro (IV-M), all three compounds (10 and 20 μM) blocked LPS binding to TLR4 without altering its expression, thereby inhibiting interleukin-1 receptor-associated kinase 1, transforming growth factor-β-activated kinase 1 (TAK1), and IKK phosphorylation and NF-κB activation. Among them, 20(S)-PPT showed the strongest anti-inflammatory activity (NC-M) [144].

Rg1 may also modulate central inflammatory responses. In a rat model of traumatic brain injury (TBI), Rg1 (5, 10, or 15 mg/kg) reduced serum IL-1β, TNF-α, and IL-6 levels. In vitro, Rg1 inhibited the interaction between non-muscle myosin IIA and heat shock protein 90 in macrophages, reducing exosomal microRNA-21 release and helping preserve BBB integrity (NC-M) [145]. In mice subjected to chronic social defeat stress, Rg1 (20 or 40 mg/kg) upregulated Sirtuin 1 (SIRT1), suppressed NF-κB transcriptional activity, and modulated MAPK signaling, as indicated by decreased p-JNK and p-p38 and increased p-ERK1/2 levels. These changes alleviated hippocampal neuroinflammation and protected neurons (NC-M) [146].

#### 3.1.2. Potential Anti-Inflammatory Mechanisms of Ginsenoside Rb1

Ginsenoside Rb1 may modulate inflammatory responses and oxidative stress. In a mouse model of colon cancer cachexia, Rb1 (10.72 mg/kg) reduced TNF-α and IL-6 levels, although the underlying molecular mechanisms remain unclear (CR-M) [147]. In mouse models of acute inflammation, sepsis, and acute kidney injury, Rb1 inhibited TLR4 dimerization and reduced TLR4–myeloid differentiation primary response 88 complex formation, thereby attenuating downstream TAK1 signaling and suppressing NF-κB and MAPK pathways. These effects were accompanied by reduced ROS accumulation and broad anti-inflammatory activity (NC-M) [148]. In rats with intestinal ischemia–reperfusion injury, intraperitoneal administration of Rb1 (15 mg/kg) enhanced antioxidant defenses via the phosphoinositide 3-kinase (PI3K)/protein kinase B (Akt)/nuclear factor erythroid 2-related factor 2 (Nrf2) pathway, reduced inflammatory cytokine levels, and ameliorated intestinal histopathological damage (NC-M) [149]. Regarding central inflammation, Rb1 (15 mg/kg) inhibited hippocampal p38 MAPK/NF-κB signaling and IDO-mediated tryptophan metabolism, thereby alleviating neuroinflammation and improving fatigue-related phenotypes in rats with postoperative fatigue syndrome (POFS) (FS-M) [150].

#### 3.1.3. Potential Anti-Inflammatory Mechanisms of Ginsenoside Rg3

Ginsenoside Rg3 primarily exerts immunomodulatory effects. In mice with LPS-induced acute lung injury, Rg3 (10, 20, or 30 mg/kg) promoted M2 macrophage polarization by activating the Mer receptor tyrosine kinase-dependent PI3K/Akt/ mechanistic target of rapamycin (mTOR) pathway, thereby suppressing pro-inflammatory mediator release and increasing anti-inflammatory cytokine levels. Similarly, in a mouse model of peritonitis, Rg3 (1 or 5 mg/kg) markedly suppressed M1 macrophage activity and reduced the release of associated inflammatory markers, including iNOS, nitric oxide, and prostaglandin E_2_. Rg3 also promoted M2 macrophage recovery and increased arginase-1 expression (NC-M) [151,152]. T-cell-related studies have shown that Rg3 suppresses the expression of the key transcription factor retinoic acid receptor-related orphan receptor γt in CD4^+^ T cells in mice with experimental autoimmune encephalomyelitis, thereby inhibiting Th17 cell differentiation and activation and alleviating autoimmune neuroinflammation (NC-M) [153]. In addition, Rg3 may alleviate immune responses and central neuroinflammation in mice with TBI by activating SIRT1, inhibiting IκBα phosphorylation, and preventing p65 nuclear translocation (NC-M) [154].

**Figure 2:**
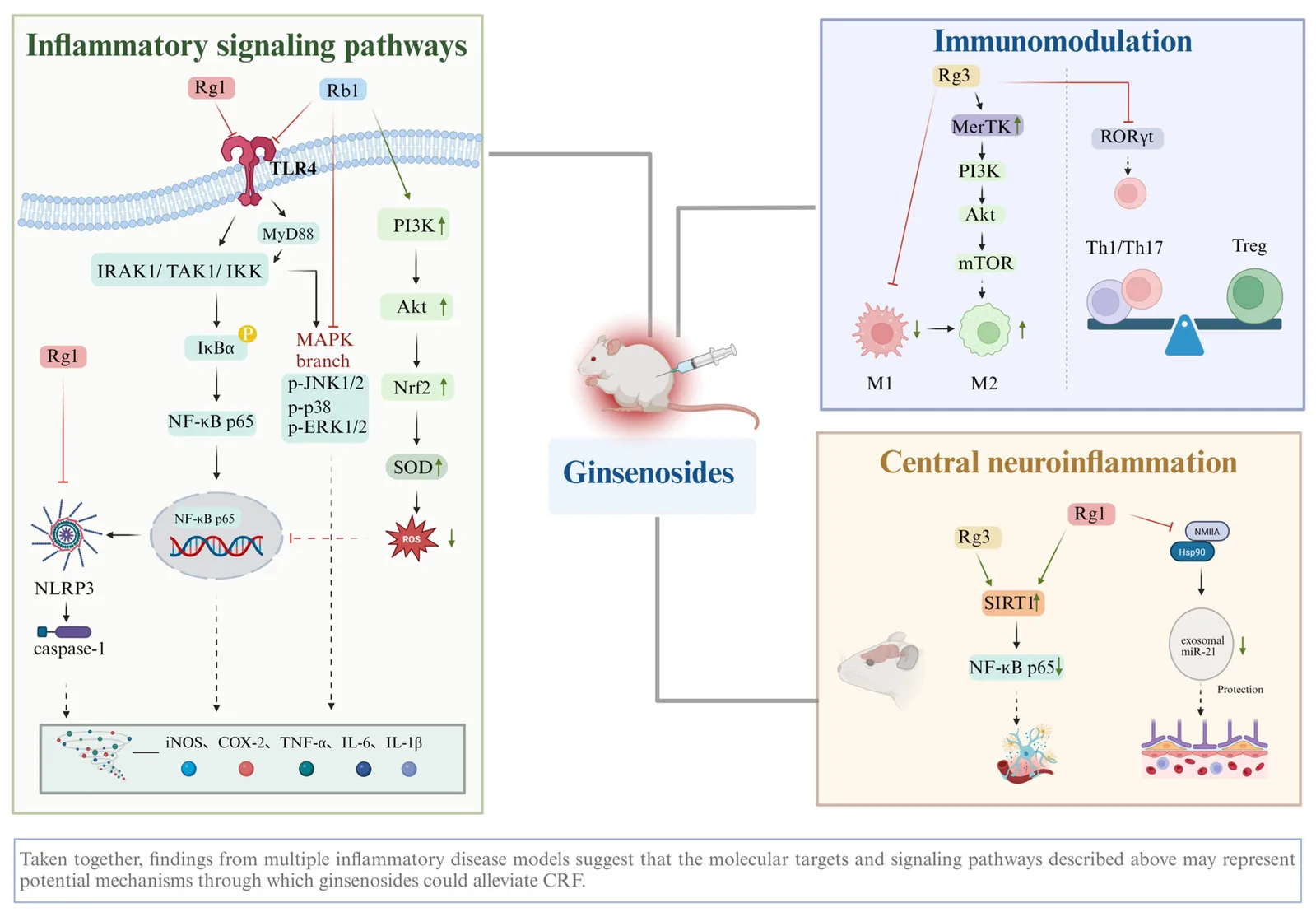
Potential anti-Inflammatory molecular mechanisms by which ginsenosides may alleviate CRF. Solid arrows indicate activation, promoting effects, or directional relationships; T-bars indicate inhibition; unidirectional dashed arrows indicate potential mechanistic effects. P, phosphorylation, small green arrows indicate increases or decreases in the corresponding parameters. Created in BioRender.

In addition to Rg1, Rb1, and Rg3, other ginsenosides may also exert anti-inflammatory effects. In a TNF-α-stimulated HepG2 cell model, Rg5, Rz1, and Rk1 inhibited NF-κB transcriptional activation. However, Rg3 did not suppress NF-κB activity in the same study, in contrast to findings from some in vivo studies of Rg3. This discrepancy highlights the heterogeneity of responses across experimental models (IV-M) [155]. Collectively, these findings suggest that ginsenosides may modulate inflammatory and oxidative stress-related pathways, providing a mechanistic basis for investigating their potential to alleviate CRF. The proposed anti-inflammatory mechanisms of ginsenosides are summarized in Figure 2.

### 3.2. Modulation of the Neuroendocrine System

Dysregulation of the HPA axis, neurotransmitter imbalances, and alterations in neuroplasticity may all contribute to the development of CRF. Current studies suggest that, in models of stress, depression, anxiety, and sleep disturbances, ginsenosides can modulate HPA axis-related hormones, monoaminergic neurotransmitters, and brain-derived neurotrophic factor (BDNF)-related signaling. These findings suggest that ginsenosides may have the potential to modulate central nervous system function.

#### 3.2.1. Potential Neuroendocrine-Regulatory Mechanisms of Ginsenoside Rg1

Ginsenoside Rg1 may regulate neuroendocrine function by modulating hormonal and neurotransmitter homeostasis. In healthy mice studied in a knockout experiment, Rg1 (12.5, 25, 50, or 100 mg/kg) inhibited the activity of lateral hypothalamic orexin neurons and activation of the paraventricular nucleus, reduced CRH and glucocorticoid levels, and improved sleep (NC-M) [156]. In animal models of stress-induced depression, Rg1 modulated the HPA and hypothalamic–pituitary–gonadal axes, upregulated GR and androgen receptor expression, and reduced serum corticosterone levels, thereby exerting stress-regulating and antidepressant effects (NC-M) [157]. These findings suggest that Rg1 may modulate negative-feedback regulation of the HPA axis. Moreover, in a mouse model of chronic restraint stress-induced depression, Rg1 (5 or 10 mg/kg) modulated multiple neurotransmitters, including 5-HT, NE, DA, acetylcholine, γ-aminobutyric acid, and glutamate. Rg1 also affected BDNF–tropomyosin receptor kinase B signaling, oxidative stress, and HPA axis regulation, thereby alleviating depressive-like behaviors and cognitive impairment induced by chronic stress (NC-M) [158].

#### 3.2.2. Potential Neuroendocrine-Regulatory Mechanisms of Ginsenoside Rb1

Ginsenoside Rb1 may help maintain monoaminergic homeostasis, although its effects vary across experimental models. In mouse and rat models of chronic unpredictable mild stress, Rb1 (4, 8, or 16 mg/kg) increased the depleted levels of 5-HT, NE, and DA, potentially by enhancing neurotransmitter synthesis through a mechanism involving the 5-HT synthesis pathway. These changes may help stabilize central neurotransmitter homeostasis, with neurotransmitter levels assessed in whole-brain tissue. In contrast, in a mouse model of acute restraint stress, Rg1 (10 mg/kg) attenuated stress-induced elevations in neurotransmitter levels in specific brain regions (NC-M) [159,160]. These findings suggest that neurotransmitter responses vary depending on the type of stressor and the brain regions examined, while Rb1 may have the potential to modulate monoamine balance.

#### 3.2.3. Potential Neuroendocrine-Regulatory Mechanisms of Ginsenoside Rg3

Ginsenoside Rg3 may also modulate neurotransmitter function. In a rat model of weight-bearing exercise-induced fatigue, Rg3 promoted tyrosine hydroxylase expression by activating the protein kinase A alpha–ERK1/2–Akt signaling pathway, improved brain DA levels, and alleviated exercise-induced fatigue (FS-M) [161]. In a rat model of stress-induced anxiety, Rg3 (20 or 40 mg/kg) ameliorated anxiety-like behaviors, attenuated HPA axis hyperactivation, and restored brain levels of the neurosteroids progesterone and allopregnanolone. Rg3 also selectively increased 5-HT levels, with no significant effects on NE or DA, suggesting coordinated regulation of neuroendocrine and neurotransmitter signaling (NC-M) [162].

**Figure 3:**
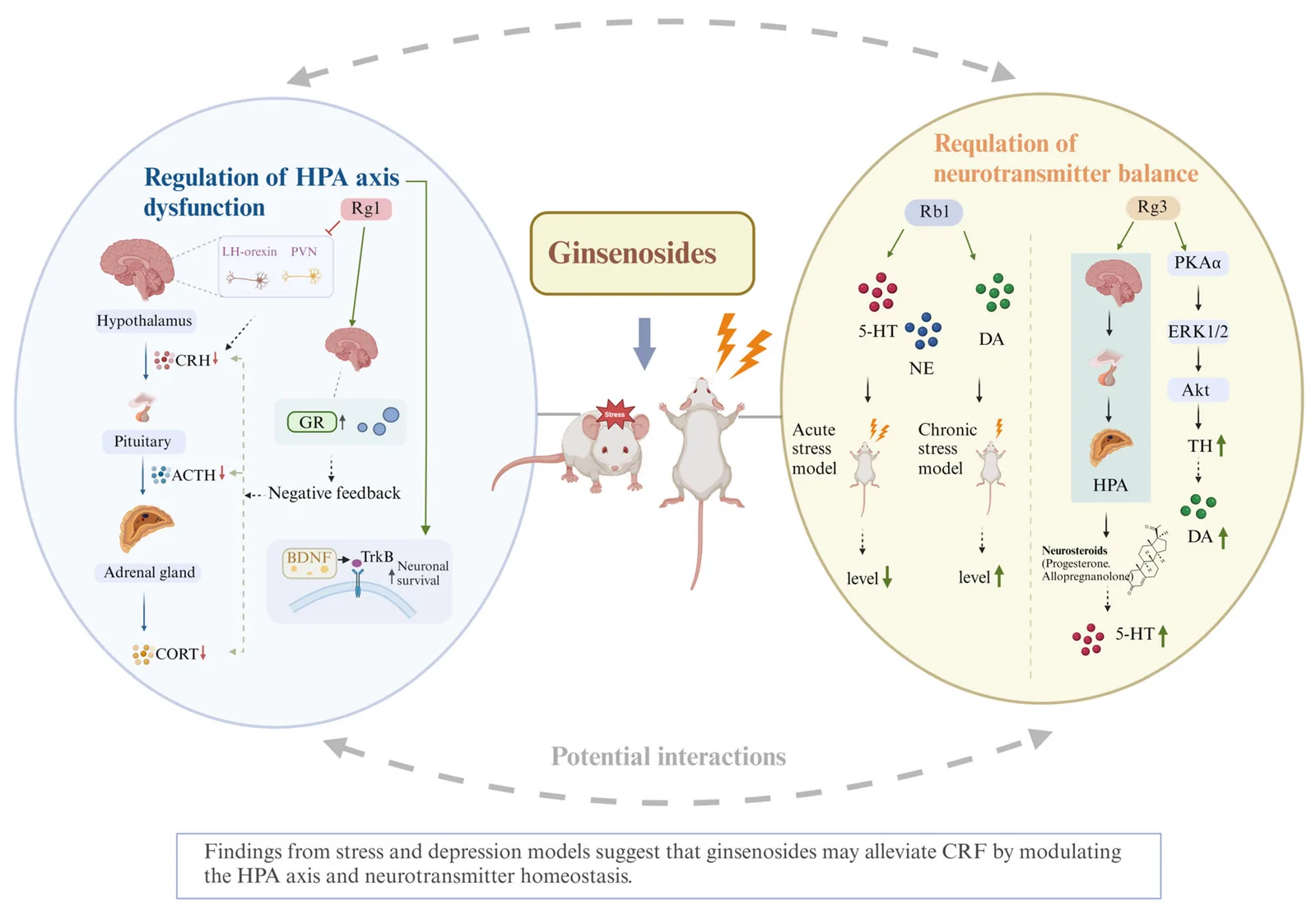
Potential molecular mechanisms through which ginsenosides may alleviate CRF by modulating neuroendocrine function. Solid arrows indicate activation, promoting effects, or directional relationships; T-bars indicate inhibition; unidirectional dashed arrows indicate potential mechanistic effects, small green arrows indicate increases or decreases in the corresponding parameters. Created in BioRender.

These findings suggest that ginsenosides may have the potential to modulate central neuroendocrine function. Further studies using CRF models are needed to clarify their effects. The potential mechanisms by which ginsenosides may modulate neuroendocrine function are illustrated in Figure 3.

### 3.3. Modulation of Skeletal Muscle Energy Metabolism

Mitochondrial damage and dysregulated oxidative stress may impair ATP supply in skeletal muscle. Energy metabolic dysfunction is an important peripheral mechanism underlying CRF. Ginsenosides may influence CRF through regulation of muscle energy metabolism; however, this hypothesis requires validation in CRF-specific models.

#### 3.3.1. Potential Energy Metabolism-Regulatory Mechanisms of Ginsenoside Rg1

In healthy C57BL/6J mice, Rg1 (0.4 mg/mL) enhanced the levels of mitochondrial OXPHOS complexes in skeletal muscle, thereby promoting oxidative metabolism. Rg1 also activated the mTOR/ ribosomal protein S6 kinase pathway to promote muscle protein synthesis and modestly downregulated the muscle atrophy marker Atrogin-1, suggesting a role in maintaining muscle protein turnover balance (NC-M) [163]. In vitro, Rg1 (0.1, 1, 10, or 100 μM) attenuated starvation-induced atrophy in C2C12 myotubes via the PI3K/Akt/mTOR/FoxO signaling pathway, suppressing ubiquitin–proteasome-mediated protein degradation involving Atrogin-1 and MuRF1. Among the tested concentrations, 10 μM showed the greatest efficacy (IV-M) [164].

#### 3.3.2. Potential Energy Metabolism-Regulatory Mechanisms of Ginsenoside Rb1

In a rat model of POFS, ginsenoside Rb1 (10 mg/kg) increased skeletal muscle Na^+^/K^+^-ATPase activity and ATP levels while maintaining succinate dehydrogenase activity (FS-M) [165]. At a dose of 15 mg/kg, Rb1 activated the PI3K/Akt/Nrf2 pathway, enhanced antioxidant defenses, and increased the levels of antioxidant enzymes, including superoxide dismutase. These effects reduced the accumulation of ROS and malondialdehyde, alleviated energy metabolic dysfunction and oxidative stress-induced damage, and improved postoperative fatigue-related symptoms (FS-M) [166]. In addition, a study using a mouse model of age-related sarcopenia found that Rb1 (20 mg/kg) suppressed aberrant NF-κB activation and promoted mitochondrial regulatory processes involving peroxisome proliferator-activated receptor gamma coactivator 1-alpha (PGC-1α), nuclear respiratory factor 1 (Nrf1), and Transcription Factor EB. These effects helped maintain mitochondrial homeostasis and attenuate age-related skeletal muscle damage (NC-M) [167].

#### 3.3.3. Potential Energy Metabolism-Regulatory Mechanisms of Ginsenoside Rg3

Ginsenoside Rg3 may exert protective effects across different cell types by promoting PGC-1α-mediated mitochondrial biogenesis and enhancing antioxidant defenses, although the upstream signaling mechanisms differ. In an injury model involving human chondrocytes, Rg3 (5, 10, or 20 μM) upregulated the SIRT1/PGC-1α/ sirtuin 3 signaling axis, reduced mitochondrial cyclophilin D acetylation, and inhibited downstream p38 MAPK/NF-κB inflammatory signaling (IV-M) [168]. In human bone marrow-derived mesenchymal stem cells, Rg3 (50 μg/mL) increased intracellular Ca^2+^ levels and activated PGC-1α-dependent mitochondrial biogenesis, thereby reducing ROS levels and cellular senescence while promoting stem cell proliferation and differentiation (IV-M) [169]. In an induced C2C12 myotube atrophy model, Rg3 activated Akt/mTOR signaling, inhibited FoxO3 transcriptional activity, suppressed the expression of muscle atrophy-related ubiquitin ligases, and promoted muscle-specific protein expression. In parallel, activation of the PGC-1α/Nrf1/mitochondrial transcription factor A pathway improved mitochondrial function and reduced oxidative damage, counteracting hormone- and inflammation-induced myotube protein degradation and atrophy. The ginseng extract BST204 exhibited similar protective effects in myotube cells (IV-M) [170,171,172].

**Figure 4:**
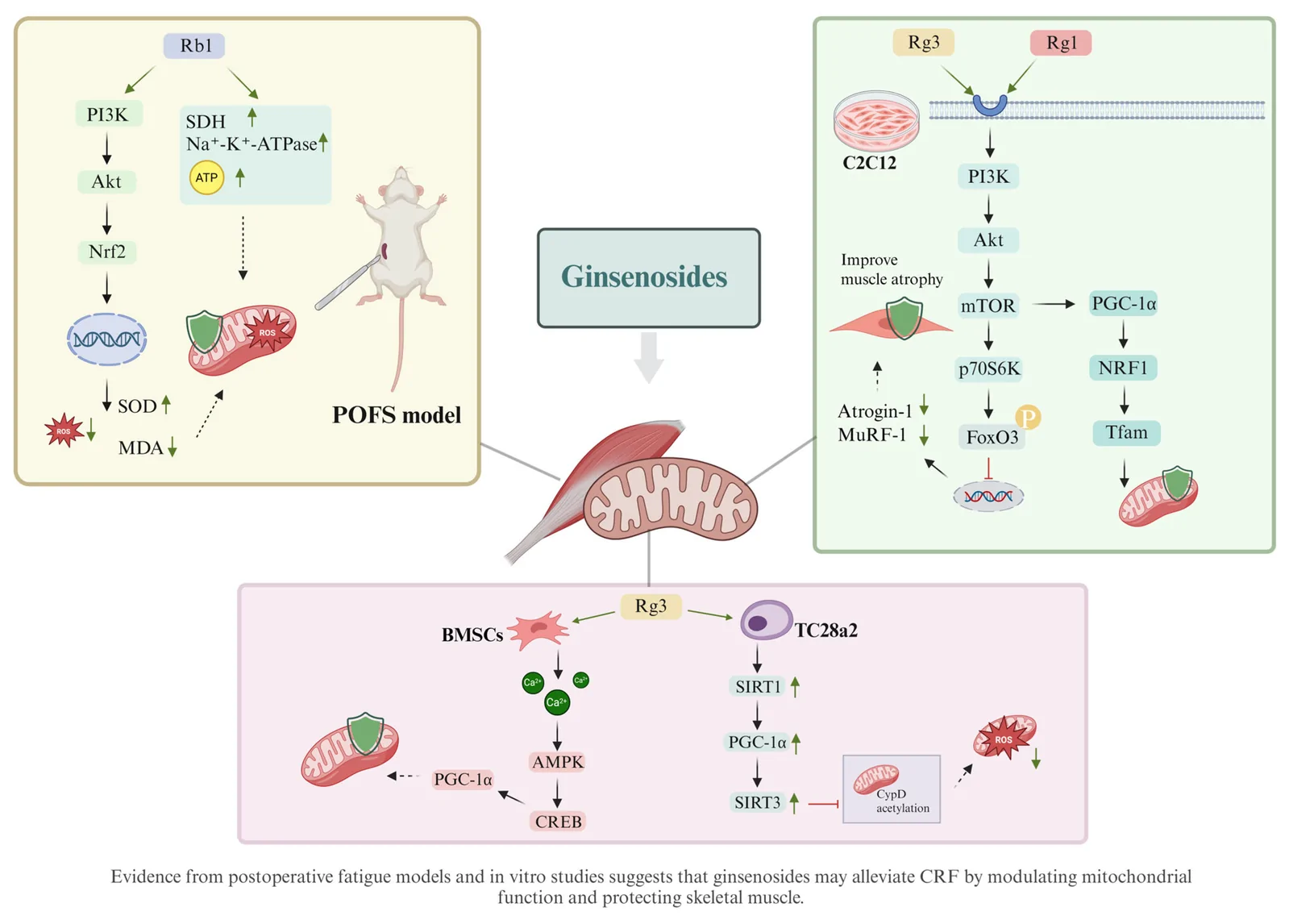
Potential molecular mechanisms through which ginsenosides may alleviate CRF by modulating skeletal muscle energy metabolism. Solid arrows indicate activation, promoting effects, or directional relationships; T-bars indicate inhibition; unidirectional dashed arrows indicate potential mechanistic effects. P, phosphorylation, small green arrows indicate increases or decreases in the corresponding parameters. Created in BioRender.

Ginsenosides may alleviate fatigue-related peripheral pathological processes by modulating mitochondrial function, oxidative stress, and skeletal muscle protein metabolism. The overlap in the signaling pathways and molecular targets involved suggests that ginsenosides may have potential regulatory effects on peripheral skeletal muscle abnormalities associated with CRF. The potential mechanisms through which ginsenosides act on skeletal muscle mitochondrial abnormalities are summarized in Figure 4.

### 3.4. Modulation of the Hematopoietic System

Beyond the mechanisms described above, existing evidence suggests that ginsenosides may also exert hematopoietic protective effects. Anemia is an important potentially modifiable contributor to CRF, and both cancer and anticancer treatments can cause bone marrow injury and hematopoietic dysfunction, thereby exacerbating fatigue. Current studies suggest that ginsenosides may protect hematopoietic function through multiple mechanisms. As no studies have directly demonstrated that ginsenosides alleviate CRF by improving anemia, this proposed pathway remains a plausible inference based on evidence supporting its individual components and requires further clinical validation. Rg1 improved hematopoietic function in mice with immune-mediated bone marrow failure by inhibiting B-cell lymphoma 2-associated X protein translocation to mitochondria (NC-M) [173]. Rb1 reduced dendritic cell overactivation by suppressing CD86 expression, modulated CD4^+^ and CD8^+^ T-cell subsets, and helped restore T-cell immune balance, thereby alleviating immune-mediated hematopoietic injury in mice (NC-M) [174]. In mouse models of cyclophosphamide (CTX) chemotherapy-induced myelosuppression, Rg1 exerts protective effects by alleviating G0/G1-phase arrest in hematopoietic cells and promoting hematopoietic recovery, primarily through the inhibition of aberrant MAPK activation (CR-M) [175]. Rg3 dose-dependently promoted the proliferation of bone marrow and splenic cells in ICR mice. In the presence of CTX, Rg3 also enhanced hematopoietic progenitor colony formation and induced the expression of mRNAs encoding hematopoiesis-related cytokines. These findings suggest that Rg3 may exert hematoprotective effects, potentially through the induction of colony-stimulating factor expression (IV-M) [176]. These findings suggest that the hematopoietic effects of ginsenosides warrant further investigation as a potential research direction in CRF, particularly in view of the contribution of anemia to fatigue.

### 3.5. Summary

Overall, the studies reviewed in this section included 11 studies on Rg1, 10 on Rb1, and 11 on Rg3, as well as two studies of ginseng extracts rich in Rg3 and Rh2 and one study of other ginsenosides. However, only one study involving the ginseng extract BST204 directly investigated the relationship between inflammatory responses and CRF. Most of the remaining studies examining the pharmacological effects of ginsenosides were conducted in models of sepsis, stress-induced depression, cachexia, postoperative fatigue, and muscle injury. This indicates that mechanistic studies directly investigating CRF remain limited. Although depression, anemia, and sleep disturbances may coexist with CRF [177,178,179], and some molecular targets and signaling pathways may overlap with those implicated in fatigue, differences in disease contexts must be considered. Therefore, whether the effects observed in other disease models also apply to CRF can only be hypothesized on the basis of shared core pathophysiological mechanisms. Available evidence suggests that ginsenosides can modulate inflammatory responses, central neuroendocrine function, skeletal muscle mitochondrial function, and hematopoietic processes. On this basis, ginsenosides may potentially alleviate CRF by acting on molecular targets and signaling pathways that overlap with those implicated in CRF pathophysiology. Nevertheless, this hypothesis requires direct validation in CRF-specific mechanistic studies.

Furthermore, most ginsenosides exhibit low oral bioavailability, and differences in their glycosylation patterns contribute to variations in oral absorption and systemic exposure. Pharmacokinetic studies have shown that, following repeated oral administration of red ginseng extract, plasma concentrations of the parent compound Rg1 remained below the lower limit of quantification (0.5 ng/mL), whereas the mean peak plasma concentrations of Rb1 and Rg3 were 12.7 ± 3.6 ng/mL and 8.7 ± 8.9 ng/mL, respectively. Less glycosylated ginsenosides and their gut microbiota-derived metabolites may achieve relatively higher systemic exposure [180,181,182]. Therefore, the translational relevance of in vitro findings should be interpreted cautiously, as the concentrations used in vitro may not reflect the systemic exposure achievable following oral administration in humans This discrepancy raises an important translational question: whether the pharmacological effects observed in preclinical studies can be achieved at the systemic exposures attainable in humans and ultimately translate into clinical benefits. In general, the pathological mechanisms discussed above provide an indirect mechanistic rationale for investigating ginsenosides as potential bioactive compounds for CRF management. Further preclinical studies and clinical investigations are needed to validate their effects on CRF and establish their translational relevance.

To clarify the experimental contexts in which the different mechanisms of the three ginsenosides discussed in this section have been investigated, the relevant studies are summarized in Supplementary Table S1.

## 4. Clinical Evidence Supporting the Effects of Ginseng in Alleviating CRF

Clinical studies suggest that ginseng preparations may provide some benefit in alleviating CRF. Among the studies included in this review, five were randomized controlled trials, one was a prospective observational study, two were pilot studies, and one was a retrospective study. The ginseng preparations investigated varied considerably, including American ginseng, Asian ginseng, red ginseng preparations, standardized extracts, and multi-herb formulations containing ginseng. The findings also differed across studies. For example, Barton et al. (2010) found that American ginseng administered at doses of 750–2000 mg/day for 8 weeks did not significantly improve the overall primary endpoint (*p* = 0.21), although signals of improvement were observed at doses of 1000–2000 mg/day [183]. In the subsequent study published in 2013, a dose of 2000 mg/day was associated with a significant improvement in the general fatigue subscale of the Multidimensional Fatigue Symptom Inventory—Short Form at week 8 (*p* = 0.003) [184]. Similarly, in the Korean red ginseng trial conducted by Kim et al., the primary Brief Fatigue Inventory endpoint was not statistically significant in the full analysis set after 16 weeks of treatment at 2000 mg/day, whereas the per-protocol analysis showed a significant improvement in fatigue [185]. Decreases in fatigue scores from baseline were also observed in a small single-arm pilot study, a small Phase II study with negative findings, and a retrospective study. In general, ginseng may have potential for alleviating CRF, but the available clinical evidence remains inconsistent and insufficient to establish a robust therapeutic effect. The studies varied in participant characteristics, dosage, treatment duration, outcome assessment tools, and preparation composition. Furthermore, most studies did not directly provide the between-group mean difference (MD) and its 95% confidence interval (CI), although they reported between-group means and p values; the MD and CI could only be derived through subsequent calculations when sufficient data were available. Consequently, despite promising efficacy signals in some studies, the current evidence does not establish a clear dose–response relationship. Moreover, the retrospective study of combination therapy by Chang et al. [186] was not a trial of ginseng monotherapy; therefore, the observed effects cannot be attributed to ginseng alone. Further studies using standardized outcome measures and comprehensive reporting of effect estimates are needed to clarify the magnitude and clinical significance of the potential benefits of ginseng for CRF.

In general, existing clinical trial evidence indicates a favorable safety profile for ginseng preparations, although a few studies have reported mild and transient adverse events. In addition, ginsenosides may affect drug transporters or related pharmacological pathways, potentially altering the efficacy and toxicity of anticancer drugs. Further research is needed to investigate potential interactions between ginseng preparations and anticancer therapies and to evaluate the safety of their concomitant use in patients with CRF. Notably, a systematic review evaluating the safety and efficacy of three types of ginseng for CRF reported improvements in CRF with American ginseng extracts containing >5% ginsenosides and Asian ginseng extracts containing ≥7% ginsenosides at certain doses. These findings suggest that ginsenoside content may be associated with the efficacy of ginseng preparations. However, across two studies by Barton et al., the reported ginsenoside content of the preparations used at effective doses decreased from 5% to 3%. This finding raises questions about the reliability of ginsenoside content as a predictor of clinical efficacy. Further studies are needed to verify this potential association. Standardization based solely on total ginsenoside content is insufficient. The concentrations and relative proportions of major individual ginsenosides, such as Rg1, Rb1, and Rg3, may vary substantially according to ginseng species, processing methods, extraction procedures, and production batches. These compounds may also exhibit distinct pharmacological properties. The clinical studies included in this review differed considerably in several aspects, including ginseng species, multi-herb formulation compositions, extraction methods, total ginsenoside content (1.49–12% or not reported), and the concentrations and relative proportions of major individual ginsenosides, such as Rg1, Rb1, and Rg3. Differences were also evident in standardization procedures, batch control, and routes of administration. These variations make direct comparisons and quantitative synthesis difficult. Furthermore, the doses of ginseng preparations used in clinical studies cannot be directly equated with those of individual ginsenosides. Most studies did not provide detailed information on ginsenoside composition. Direct evidence demonstrating that individual ginsenosides can confer the same benefits for CRF as whole ginseng preparations remains lacking. In addition, one study investigated a multi-herb formulation containing ginseng. The observed antifatigue effects may have resulted from the combined actions of multiple constituents, because the preparation also contained other medicinal herbs. Therefore, these effects cannot be directly attributed to ginseng or ginsenosides alone. This limitation should also be considered when interpreting the clinical evidence. Future studies should use ginseng preparations with clearly defined compositions and standardized quality criteria, report detailed ginsenoside content, and place greater emphasis on quantitative effect measures. These improvements would facilitate a more accurate assessment of the clinical benefits of ginseng interventions. A summary of the study designs and intervention protocols of clinical studies investigating ginseng for CRF is presented in Table 4, with comprehensive tables covering all included studies provided in Supplementary Materials Tables S2 and S3.

## 5. Conclusions and Future Perspectives

Rg1, Rb1, and Rg3 are among the most extensively investigated representative ginsenosides. Accordingly, this review further synthesizes the preclinical evidence regarding their potential mechanisms of action. Current findings suggest that these three ginsenosides may influence several pathological processes associated with CRF, including inflammatory responses, neuroendocrine regulation, energy metabolism, and hematopoietic function. Their potential mechanisms may involve inflammatory signaling pathways, such as TLR4/NF-κB and MAPK, modulation of HPA axis-related hormones, and regulation of skeletal muscle protein metabolism. However, most mechanistic studies have been conducted in models of inflammation, stress, or chemotherapy-induced injury rather than in standardized tumor-associated CRF models. Therefore, these findings primarily suggest potential mechanistic associations and should not be considered direct evidence of efficacy in CRF models or patients. The three ginsenosides also appear to differ in their predominant pharmacological activities. As discussed in this review, Rg1 and Rb1 appear to primarily modulate inflammation-related signaling pathways, whereas Rg3 appears to have more prominent immunomodulatory effects. Regarding neuroendocrine regulation, Rg1 has been associated with modulation of HPA axis activity and related hormone levels, while Rb1 and Rg3 have also demonstrated regulatory effects on neurotransmitter homeostasis. Rg3 may additionally influence HPA axis activity and neurosteroid levels. These findings suggest that individual ginsenosides may differ in their predominant mechanistic effects, although their activities may overlap. However, these differences do not establish the superiority of any particular ginsenoside in terms of clinical efficacy for CRF. Consequently, future head-to-head comparative studies are warranted to clarify the relative effects of individual ginsenosides on CRF-related outcomes.

CRF has attracted increasing attention in oncology in recent years. TCM and natural bioactive compounds have shown potential as research candidates for the comprehensive management of CRF. From a clinical perspective, the current evidence supporting the use of ginseng for CRF is derived primarily from studies of standardized ginseng extracts and ginseng-containing preparations. The findings suggest that ginseng may provide some relief from CRF; however, the available evidence remains inconclusive because of heterogeneity across studies. The efficacy of ginseng preparations may be associated with the composition and standardization of their bioactive constituents, particularly ginsenosides. Nevertheless, further experimental and clinical studies are needed to clarify the independent contributions of individual ginsenosides, their potential synergistic interactions with other constituents, and the optimal dosing regimens. The limitations of current research on ginseng and ginsenosides for alleviating CRF are summarized in Table 5.

Future research should first prioritize the comprehensive characterization of the chemical composition of ginseng preparations—especially the identification and quantification of major ginsenosides—as well as the standardization of the preparations themselves. Building on this foundation, studies using standardized CRF models should be prioritized to clarify the relationships between the molecular targets of ginsenosides and fatigue-related outcomes, as well as to determine the doses required to achieve relevant systemic exposure. Mechanistic investigations should integrate network pharmacology and multi-omics approaches to identify key molecular targets and signaling pathways, followed by experimental validation in CRF models. To address the limited oral bioavailability of certain ginsenosides, novel drug delivery strategies, such as nanoformulations and liposomes, could be explored to improve bioavailability and optimize their pharmacological effects. From a clinical perspective, large-scale, multicenter, randomized, double-blind, controlled trials are needed. These trials should use ginseng preparations with well-characterized compositions and standardized quality criteria to establish effective doses and treatment durations and to determine the clinical significance of observed changes. Outcomes should be reported using standardized effect measures, including MDs, 95% CIs, and effect sizes. In addition, CRF-related biomarkers, including inflammatory cytokines, oxidative stress markers, indicators of mitochondrial function, and measures of energy metabolism, could be incorporated to investigate the relationships between clinical outcomes and potential underlying mechanisms. Overall, the available evidence supports further investigation of ginseng and ginsenosides as potential adjunctive interventions for CRF. With continued advances in preparation standardization, mechanistic elucidation, and the accumulation of high-quality clinical evidence, ginseng and its bioactive constituents may offer new research directions for the comprehensive management of CRF.

## Figures and Tables

**Table 1:** Comparison of ginsenosides in different *Panax* species and their processed products.

	Asian Ginseng	American Ginseng	Red Ginseng	Black Ginseng
Original species	Panax ginseng	Panax quinquefolius	Panax ginseng (processed from Asian ginseng)	Panax ginseng (processed from Asian ginseng)
Origin	Native to East Asia in the Northern Hemisphere; primarily cultivated in East Asia, with small-scale cultivation in Northern Europe.	Temperate regions of North America, extending southward from Quebec, Canada, to Georgia, USA.	The Republic of Korea, as well as ginseng-producing regions in China, including the Changbai Mountain area in Jilin Province and Liaoning Province.	Initially developed in Korea and subsequently widely used in China and Southeast Asia.
Processing method	Fresh ginseng roots are peeled and dried directly.	Fresh ginseng roots are peeled and dried directly.	Fresh ginseng roots are steamed at approximately 100 °C and then dried.	Repeated steaming and sun-drying (nine cycles).
Representative major ginsenosides	Such as Rb1, Rb2, Rc, Rd, Re, Rg1, Rf	Such as Rb1, Re, Rd, F11	Such as Rg3, Rh1, Rk1, Rg5, Rh2	Such as Rg3, Rh1, Rk1, Rg5, Rh2, Rk3, Rh4

The ginsenoside content and profiles of different Panax species and their processed products vary with cultivation age, geographical origin, and the extraction and analytical methods used. Red ginseng and black ginseng are processed forms of Panax ginseng; they are not independent species. The major ginsenosides listed are representative rather than exhaustive. Data from [51,52,53].

**Table 2:** Evidence classification of sources supporting CRF pathogenesis.

Evidence Source	Relevance to CRF	Abbreviation Used in This Review
Human CRF studies	Direct clinical evidence	H-CRF
Cancer-induced fatigue models	Direct/preclinical CRF-related evidence	C-IF
Chemotherapy-induced fatigue models	Treatment-related fatigue evidence	CH-IF
Cancer cachexia models	Indirect mechanistic evidence	CC
Non-cancer fatigue models	Indirect evidence	NC-F
In vitro studies	Mechanistic evidence	IV

Review articles were excluded from the evidence grading. Evidence grading was restricted to the section on CRF pathogenesis.

**Table 3:** Evidence grading of studies on the potential of ginsenosides to alleviate CRF.

Evidence Source	Relevance to CRF	Abbreviation Used in This Review
CRF models	Direct evidence for CRF	C-M
Non-CRF models	Indirect mechanistic evidence	NC-M
Fatigue-specific models	Fatigue-specific evidence	FS-M
Cancer-related models	Indirect evidence from cancer-related models	CR-M
In vitro models	Indirect mechanistic evidence	IV-M

Evidence grading was restricted to studies investigating the models used to evaluate the potential mechanisms of action of ginsenosides.

**Table 4:** Concise summary of clinical studies of ginseng for CRF.

First Author, Year	Sample/Study Design	Ginseng Preparation	Control	Main Outcomes and Results	Safety	Preliminary Evidence Level
Barton et al. (2010) [183]	Randomized, double-blind, placebo-controlled, dose-finding pilot study; 290 participants enrolled, 282 randomized, and 175 completed the study; 8 weeks	American ginseng (approximately 5% ginsenosides); 750, 1000, or 2000 mg/day.	Placebo	overall ginseng vs. placebo (BFI, *p* = 0.21); a nonsignificant trend was observed in the high-dose group. MD/CI NR (arm means, arm 95% CIs, effect sizes, and *p* values were reported).	No significant between-group differences in toxicity	1b (Medium)
Barton et al. (2013) [184]	Multicenter, randomized, double-blind, placebo-controlled RCT (364 participants randomized; 341 initiated treatment); 8 weeks	American ginseng (approximately 3% ginsenosides); 2000 mg/day.	Placebo	(MFSI-SF at week 4, *p* = 0.07); however, a secondary analysis at week 8 repeated analysis showed a significant difference (*p* = 0.003). MD/CI NR (6.2/9.7 are calculated values).	No apparent differences in toxicity between groups	1b (Low)
Yennurajalingam et al. (2015) [187]	Prospective, open-label, single-arm pilot study (30 participants treated; 24 evaluable); 29 days	Standardized Asian ginseng extract (7% ginsenosides and malonyl ginsenosides; actual content, 12% ± 3%); 800 mg/day.	None	(FACIT-F, *p* < 0.001); 87% at day 15 of participants experienced an improvement of ≥3 points. Not applicable (single-arm study with no between-group comparison).	No Grade ≥ 3 drug-related adverse events reported	4 (Low)
Yennurajalingam et al. (2017) [188]	Randomized, double-blind, placebo-controlled RCT (127 participants randomized; 112 evaluable); 28 days	Standardized Asian ginseng extract (7% ginsenosides and malonyl ginsenosides); 800 mg/day.	Placebo	(FACIT-F, *p* = 0.67); both groups improved, with no significant between-group difference. MD/CI NR (1.0 is a calculated value).	Fewer any-grade toxicities with ginseng; one severe infection reported.	1b (Medium)
Pourmohamadi et al. (2018) [189]	Prospective, randomized, placebo-controlled study (blinding not reported; 54 participants in the ginseng group and 60 in the control group); 30 days	Asian ginseng; 100 mg/day; ginsenoside content NR.	Placebo	(BEK/Beck, *p* < 0.0001 (between-group P not reported)); improvements in depression and quality of life (QoL) were reported. MD/CI NR (percentages and *p* values only).	Insufficient adverse event reporting	1b (Low)
Chang et al. (2018) [186]	Single-center retrospective chart review (28 participants received combination therapy; 15 completed the study); mean duration of 30.5 days	American ginseng; 2000 mg/day; methylphenidate (10–40 mg/day) as combination therapy; ginsenoside content NR.	No control group	(ESAS, *p* < 0.00002); 60% of participants experienced an improvement of ≥3 points. Not applicable (no control group).	Three Grade 2 adverse events reported	4 (Low)
Kim et al. (2020) [185]	Multicenter, randomized, double-blind, placebo-controlled RCT (438 participants randomized; 409 included in the full analysis set); 16 weeks	Korean red ginseng (total content of seven ginsenosides: 1.49% ± 0.4%, reported in Data Supplement 3, not main text); 2000 mg/day.	Placebo	(BFI in the full analysis set [FAS], *p* = 0.119); statistically significant in the per-protocol (PP) set (*p* = 0.019). Partially reported (not for the primary endpoint; subgroup result for participants aged ≥60 years: 6.14 [2.20, 10.45]).	Similar overall adverse event rates; higher incidence of neutropenia	1b (Medium)
Guglielmo et al. (2020) [190]	Randomized, double-blind, placebo-controlled trial (32 participants randomized: 17 in the ginseng group and 15 in the control group); 8 weeks	American ginseng (Equunax^®^); 1000 mg/day; ginsenoside content NR.	Placebo	(BFI at week 8, *p* = NS); one individual activity-interference item favored placebo. MD/CI NR (0.58 is a calculated value).	Insufficient adverse event reporting	1b (Medium)
Wang et al. (2024) [191]	Prospective, multicenter, nonrandomized, open-label, controlled cohort study (529 participants allocated to groups; 409 included in the final analysis); 8 weeks	Renshen Guben Oral Liquid; 10 mL twice daily (20 mL/day); ginsenoside content NR; multi-herb formulation.	Supportive care	(MFI at week 8, *p* < 0.001), week 4 was not significant (*p* = 0.153); improvements in BFI and FACT-Hep were also reported. Fully reported (e.g., BFI effect estimate: −1.73 [−2.26, −1.20]).	Similar rates of overall and Grade ≥ 3 adverse events	2b (Low)

RCT, randomized controlled trial; BFI, Brief Fatigue Inventory; MFSI-SF, Multidimensional Fatigue Symptom Inventory–Short Form; FACIT-F, Functional Assessment of Chronic Illness Therapy—Fatigue; ESAS, Edmonton Symptom Assessment System; BEK/Beck, Beck assessment (instrument name as reported in the original study); FACT-Hep, Functional Assessment of Cancer Therapy—Hepatobiliary; QoL, quality of life; MD, mean difference; CI, confidence interval; NR, data not explicitly reported in the original article; NS, not statistically significant; FAS, full analysis set; PP, per-protocol population. Evidence levels were assigned according to study design using the 2011 criteria of the Oxford Centre for Evidence-Based Medicine (CEBM). When evidence levels were not reported in the original articles, they were assigned based on study design. Evidence levels reflect the methodological hierarchy of study designs and should not be interpreted as equivalent to the overall certainty of evidence or the reliability of treatment effects.

**Table 5:** Limitations of current research on ginseng and ginsenosides for alleviating CRF.

Study Type	Current Status
Clinical studies of ginseng preparations	Limited high-quality evidence; inconsistent study findings; substantial heterogeneity in preparations; inadequate standardization and quality control; inconsistent doses, treatment durations, and outcome measures; incomplete reporting of effect sizes; confounding by multi-herb formulations, precluding attribution of effects to ginseng alone; unclear relationship between ginsenoside content and efficacy; incomplete safety data.
Potential molecular mechanisms of ginsenosides	Limited mechanistic studies directly investigating CRF; evidence largely provides indirect mechanistic insights; low oral bioavailability and a mismatch between in vitro effective concentrations and human systemic exposure; insufficient preclinical-to-clinical translational validation; heterogeneity in experimental models.

## Data Availability

No new data were created or analyzed in this study. Data sharing is not applicable to this article.

## Supplementary Materials

The following supporting information can be downloaded at https://www.mdpi.com/article/10.3390/molecules31213917/s1: Table S1: Summary of the potential mechanisms by which ginsenosides may alleviate CRF; Table S2: Study Designs and Intervention Protocols of Existing Clinical Studies Investigating Ginseng for CRF.; Table S3: Clinical findings, limitations, and evidence assessment of ginseng for CRF.

## Abbreviations

CRF: Cancer-related fatigue; TCM, Traditional Chinese medicine; PPT, protopanaxatriol; HPA, hypothalamic–pituitary–adrenal; ATP, adenosine triphosphate; TNF-α, tumor necrosis factor-α; IL-1β, interleukin-1β; IL-6, interleukin-6; IκB, inhibitor of κB; NF-κB, nuclear factor-κB; IKK, IκB kinase; NLRP3, NOD-, LRR- and pyrin domain-containing protein 3; ROS, reactive oxygen species; JAK, Janus kinase; STAT3, signal transducer and activator of transcription 3; UPS, ubiquitin–proteasome system; TLR4, Toll-like receptor 4; PIF, including proteolysis-inducing factor; CNS, central nervous system; BBB, blood–brain barrier; GR, glucocorticoid receptor; LPS, lipopolysaccharide; CRH, corticotropin-releasing hormone; 5-HT, 5-hydroxytryptamine; DA, dopamine; NE, norepinephrine; IDO, indoleamine 2,3-dioxygenase; TRP, tryptophan; ME/CFS, myalgic encephalomyelitis/chronic fatigue syndrome; mtDNA, mitochondrial DNA; OXPHOS, oxidative phosphorylation; FoxO, forkhead box O; MuRF1, muscle RING finger protein 1; MAPK, mitogen-activated protein kinase; TAK1, transforming growth factor-β-activated kinase 1; Th17, T helper 17; iNOS, inducible nitric oxide synthase; TBI, traumatic brain injury; SIRT1, sirtuin 1; ERK, extracellular signal-regulated kinase; PI3K, phosphatidylinositol 3-kinase; Akt, protein kinase B; Nrf2, nuclear factor erythroid 2-related factor 2; POFS, postoperative fatigue syndrome; MerTK, Mer receptor tyrosine kinase; mTOR, mammalian target of rapamycin; BDNF, brain-derived neurotrophic factor; Nrf1, nuclear respiratory factor 1; SIRT1, Sirtuin 1; PGC-1α, peroxisome proliferator-activated receptor gamma coactivator 1-alpha; CTX, cyclophosphamide; MDs, mean differences; CIs, confidence intervals; H-CRF, human cancer-related fatigue studies; C-IF, cancer-induced fatigue models; CH-IF, chemotherapy-induced fatigue models; CC, cancer cachexia models; NC-F, non-cancer fatigue models; IV, in vitro studies; C-M, cancer-related fatigue models; NC-M, non-cancer-related fatigue models; FS-M, fatigue-specific models; CR-M, cancer-related models; IV-M, in vitro models; cAMP, cyclic adenosine monophosphate; PKA, protein kinase A; CREB1, cAMP response element-binding protein 1.
