# Supplementary material for: Potential Molecular Mechanisms of Ginsenosides for Alleviating Cancer-Related Fatigue and Clinical Research Progress of Ginseng Preparations: A Review
Source: Molecules. 2026 Oct 30;31(21):3917. doi: 10.3390/molecules31213917 (PMC13764966; doi:10.3390/molecules31213917)
Supplement: Supplementary file 1 [file molecules-31-03917-s001.zip › molecules-4617986-supplementary/Supplementary Tables(S1–S3).pdf]

The supplementary tables (Tables S1–S3) are provided below:

Table S1. Summary of the Potential Mechanisms by Which Ginsenosides May Alleviate CRF.

Compound/Dose	Experimental model	Mechanistic focus	Key Molecular Targets and Pathways	Evidence classification	Evidence level
BST204 (Rg3 10.9% + Rh2 7.2%; mixture); 100/200 mg/kg, p.o., for 28 days	5-FU-induced CRF in tumor-bearing mice	Inflammation	TNF-α and IL-6	Direct behavioral evidence in an animal CRF model	Moderate [142]
Rg1; 20 μM in vitro; in vivo dose not specified	LPS-induced sepsis-induced myocardial dysfunction in mice; LPS-stimulated neonatal rat cardiomyocytes	Inflammation	TLR4/NF-κB/NLRP3 signaling axis	Indirect mechanistic evidence	Low to moderate [143]
Rg1; 10–20 μM in vitro; Rg1 20 mg/kg p.o. in vivo	LPS-stimulated peritoneal macrophages; TNBS-induced colitis in mice	Inflammation	Inhibits LPS–TLR4 binding; decreases p-IRAK1, p-TAK1, p-IKKβ, p-IκBα, and p-p65; inhibits NF-κB nuclear translocation; modulates the Th17/Treg balance	Indirect mechanistic evidence	Low to moderate [144]
Rg1; 5/10/15 mg/kg, i.p., in rats, administered 30 min and 24 h after injury; 1 μmol/L in vitro	Traumatic brain injury (TBI) in rats; IL-1β-stimulated THP-1 macrophages; human brain microvascular endothelial cells	Neuroinflammation/blood–brain barrier	Inhibits macrophage-derived exosomal miR-21 release via the NMIIA–HSP90 pathway; increases TIMP3; decreases MMP-1/3/9; preserves ZO-1, occludin, and claudin-5; inhibits NF-κB p65 phosphorylation	Indirect mechanistic evidence	Low to moderate [145]
Rg1; 20/40 mg/kg, i.g., for 34 days	Chronic social defeat stress-induced depression in mice	Neuroinflammation	Inhibits microglial activation (Iba1↓); decreases IL-6, TNF-α, IL-1β, iNOS, COX-2, cleaved caspase-3/9, p-p65, and ac-p65; increases SIRT1; modulates MAPK signaling (p-JNK↓, p-p38↓, p-ERK↑)	Indirect mechanistic evidence	Low to moderate [146]
Rb1 (with WEG, GE5, and GE50 as comparators); 10.72 mg/kg, orally, for 23 days	C26 colon cancer cachexia in mice	Inflammation	Decreases serum TNF-α and IL-6	Indirect evidence from cancer-related models	Moderate [147]
Rb1; 10/20/40 μM in vitro; 5/10/20 mg/kg, i.p., in vivo	LPS-stimulated RAW264.7 cells, bone marrow-derived macrophages, and THP-1 cells; LPS-/CTD-induced acute kidney injury in mice/rats; LPS-induced sepsis mortality; xylene-induced ear edema	Inflammation	Blocks TLR4 dimerization by targeting TLR4–MD2; inhibits MyD88–TAK1 signaling; decreases IKKα/β, IκBα, and p65 phosphorylation; decreases JNK1/2 and p38 phosphorylation, Ca^{2+}, ROS, NO, iNOS, and COX-2	Indirect mechanistic evidence	Low to moderate [148]
Rb1; 15 mg/kg, i.p., 1 h before surgery	Intestinal ischemia–reperfusion injury in rats	Inflammation/oxidative stress	Activates PI3K/Akt/Nrf2 signaling; increases p-p85, p-Akt, and Nrf2; decreases MDA and 8-iso-PGF2α; increases SOD	Indirect mechanistic evidence	Low to moderate [149]
Rb1; 15 mg/kg, i.p., from 3 days before surgery until sacrifice	Postoperative fatigue syndrome (POFS) in rats (70% small intestine resection)	Neuroinflammation	Inhibits p38 MAPK/NF-κB/p65 and IDO signaling; decreases hippocampal IL-1β, IL-6, and TNF-α; reduces the kynurenine/tryptophan ratio	Fatigue-specific evidence	Moderate [150]

Compound/Dose	Experimental model	Mechanistic focus	Key Molecular Targets and Pathways	Evidence classification	Evidence level
Rg3 monotherapy; 10/20/30 mg/kg, i.p., in vivo; 25/50/100 μg/mL in vitro	LPS-induced acute lung injury in wild-type and MerTK$^{-/-}$ mice; LPS-stimulated RAW264.7 cells	Inflammation/ immunomodulation	Activates MerTK-dependent PI3K/Akt/mTOR signaling; decreases MPO, TNF-α, IL-1β, and IL-6; increases IL-10 and TGF-β; promotes M2 macrophage polarization	Indirect mechanistic evidence	Low to moderate [151]
Rg3 monotherapy; 1–10 μM in vitro; 1 or 5 mg/kg, i.p., in vivo, administered after the peak of inflammation	LPS-stimulated mouse peritoneal macrophages; zymosan-induced peritonitis in C57BL/6 mice	Inflammation/ immunomodulation	Promotes M2 polarization mainly by preserving/restoring arginase-1; IL-10, TGF-β, and Ym-1 were not significantly changed; suppresses M1 polarization (iNOS↓, NO↓, PGE_2↓; COX-2 protein inhibition not significant)	Indirect mechanistic evidence	Low to moderate [152]
Rg3 monotherapy; 18.75/37.5 μg/mL in vitro; T cells treated ex vivo before adoptive transfer	In vitro dendritic cell–T cell co-culture and dendritic cell-free Th17 differentiation systems; experimental autoimmune encephalomyelitis in mice following adoptive transfer of myelin oligodendrocyte glycoprotein-reactive T cells	Neuroinflammation/immunomodulation	Inhibits Th17 differentiation (IL-17A, RORγt, IL-21, and IL-22↓) through T-cell-intrinsic mechanisms; has limited effects on dendritic cell secretion of IL-6, TNF-α, and IL-12p40	Indirect mechanistic evidence	Low to moderate [153]
Rg3 monotherapy; 5/10 mg/kg, i.p., 4 h after surgery and on days 1–7 postoperatively; 1–20 μM in vitro	TBI in mice; LPS-stimulated primary microglia; hippocampal neurons	Neuroinflammation	Activates SIRT1 and inhibits NF-κB; decreases p-IκBα, iNOS, and nuclear p65; reduces Iba1/iNOS colocalization in microglia	Indirect mechanistic evidence	Low to moderate [154]
Ginsenosides Rg5, Rz1, Rk1, Rg3, Rb1, and others; 0.1/1/10 μM	TNF-α-stimulated HepG2 cells	Inflammation	NF-κB luciferase reporter activity; COX-2 and iNOS mRNA expression	Indirect mechanistic evidence	Low [155]
Rg1; 12.5/25/50/100 mg/kg, i.p., for 4 consecutive days	Sleep–wake monitoring in C57BL/6J mice; ablation of orexin neurons in the lateral hypothalamus and corticotropin-releasing hormone neurons in the paraventricular hypothalamus	Neuroendocrine regulation/sleep	Inhibits CRH neurons in the paraventricular hypothalamus (PVH) and orexin neurons in the lateral hypothalamus (LH), as indicated by decreased c-Fos; sleep architecture assessed by EEG/EMG	Indirect mechanistic evidence	Low to moderate [156]
Rg1; acute treatment at 5/10/20 mg/kg, i.g.; CUMS model, 5/10/20 mg/kg, i.g., for 28 days; GDX model, 10/20/40 mg/kg, i.g.	Forced swim test and tail suspension test in mice; chronic unpredictable mild stress (CUMS)-induced depression in rats; gonadectomy-induced neuroendocrine dysfunction in mice	Neuroendocrine regulation	HPA axis: decreases serum corticosterone and increases glucocorticoid receptor (GR) expression in the hippocampus/PFC. HPG axis: increases serum testosterone and androgen receptor (AR) expression in the PFC	Indirect mechanistic evidence	Low to moderate [157]
Rg1; 5/10 mg/kg, i.p., once daily starting 14 days after stress exposure	Chronic restraint stress-induced depression in rats	Neuroendocrine regulation	HPA axis: decreases serum corticosterone (CORT). Neurotransmitters: increases PFC 5-HT, 5-HIAA, and GABA and decreases Glu; NE and ACh were not significantly increased. BDNF/TrkB signaling: increases	Indirect mechanistic evidence	Low to moderate [158]

Compound/Dose	Experimental model	Mechanistic focus	Key Molecular Targets and Pathways	Evidence classification	Evidence level
			BDNF, TrkB, and Bcl-2 and decreases Bax		
Rb1; 4/8/16 mg/kg, p.o.	FST and TST in mice; 5-hydroxytryptophan-induced head-twitch test and reserpine antagonism test; CUMS in rats	Neurotransmitter homeostasis	Central monoaminergic neurotransmitters: increases brain 5-HT, 5-HIAA, NE, and DA in CUMS rats; reverses reserpine-induced ptosis, hypothermia, and reduced locomotor activity; enhances the 5-HTP-induced head-twitch response	Indirect mechanistic evidence	Low to moderate [159]
Rb1; 10 mg/kg, p.o., 30 min before stress exposure	Acute restraint stress in mice (30 min)	Neurotransmitters/fatigue	Frontal cortex and cerebellar monoamines: DOPA, DA, NE, EP, DOPAC, 5-HT, 5-HIAA, and 5-HTP. Previous studies cited in the article reported decreased plasma corticosterone, but HPA-axis hormones were not directly measured in the present study	Indirect mechanistic evidence	Low to moderate [160]
Rg3 monotherapy; 10/50/100 mg/kg, i.g., before swimming; 0.1–100 μM in vitro	Weight-loaded forced swimming fatigue in rats; SH-SY5Y cells	Neurotransmitter homeostasis	Central dopaminergic system: increases TH expression and p-Ser40 TH, PKAα, p-ERK1/2, p-Akt, and DA; modulates α-synuclein; decreases the DOPAC/DA ratio	Fatigue-specific evidence	Moderate [161]
Rg3 monotherapy; 10/20/40 mg/kg, i.g., once daily from days 29–43	Chronic unpredictable stress-induced anxiety in rats	Neurotransmitters/neuroplasticity	HPA axis: decreases serum corticosterone (Cort), CRH, and ACTH. Neurosteroids: increases progesterone and allopregnanolone in the PFC and hippocampus. Serotonergic system: increases 5-HT in the PFC and hippocampus; no significant effects on DA, NE, DOPAC, or HVA	Indirect mechanistic evidence	Low to moderate [162]
Rg1; 0.4 mg/mL in drinking water for 5 weeks (5 weeks per abstract/methods, but 4 weeks in Results)	Three-month-old C57BL/6J mice; primary myoblasts	Energy metabolism	Oxidative metabolism: increases NADH-TR and SDH activity, OXPHOS complexes I–V, COX2, SDHB, MB, GLUT4, HK, PK, and PFK. Anabolic signaling: increases p-S6K and increases p-4EBP1 and decreases total 4EBP1; increases MHC I, IIB, and IIX expression; increases CSA of MHC IIA-, IIB-, and IIX-positive fibers, muscle cross-sectional area (CSA), and grip strength	Indirect mechanistic evidence	Low to moderate [163]
Rg1; 10^{-4}–10^{-1} mM (48 h for viability; 5–60 min and 24 h for signaling)	C2C12 myotubes; starvation-induced atrophy	Skeletal muscle protein metabolism	Inhibition of protein degradation: decreases Atrogin-1 and MuRF1. Akt/mTOR/FOXO signaling: increases p-Akt, p-mTOR, and p-FOXO1/3a; effects are reversed by LY294002	Indirect mechanistic evidence	Low to moderate [164]
Rb1; 10 mg/kg, i.p.	POFS in rats induced by major small intestinal resection	Energy metabolism	Increases skeletal muscle ATP levels, Na^+/K^+-ATPase activity, and SDH activity; no significant change in PK activity	Fatigue-specific evidence	Moderate [165]
Rb1; 15 mg/kg, i.p., from 3 days before surgery until sacrifice	POFS in aged rats (70% small intestine resection)	Oxidative stress	Skeletal muscle oxidative stress: increases SOD and decreases ROS and MDA. PI3K/Akt/Nrf2 signaling: increases p-Akt, Nrf2	Fatigue-specific evidence	Moderate [166]

Compound/Dose	Experimental model	Mechanistic focus	Key Molecular Targets and Pathways	Evidence classification	Evidence level
			nuclear translocation, and Akt2/Nrf2 mRNA expression		
Rb1; 20/40 μM in vitro; 20 mg/kg, i.p., in vivo for 4 weeks	H_2O_2-treated C2C12 myoblasts; aged mice	Mitochondrial homeostasis	Mitochondrial function: increases mitochondrial membrane potential (MMP) and ATP and decreases cytochrome c (CytC) release. Mitochondrial biogenesis: increases Nrf1, PGC-1α, TFEB, Tom20, and mtDNA. Decreases ROS, apoptosis, and NF-κB signaling	Indirect mechanistic evidence	Low to moderate [167]
Rg3 monotherapy; 5–20 μM in vitro	Human chondrocytes (TC28a2 cells) stimulated with TNF-α	Mitochondrial function	Mitochondrial function: increases mtDNA content, mitochondrial mass, and MMP and decreases ROS; modulates the SIRT1/PGC-1α/SIRT3 axis; inhibits p38 MAPK/NF-κB signaling	Indirect mechanistic evidence	Low [168]
Rg3 monotherapy; 1–50 μg/mL in vitro for 5 days	Human bone marrow-derived mesenchymal stem cells	Mitochondrial function	Mitochondrial biogenesis: increases PGC-1α, TFAM, SDHB, and UQCRC2; increases OCR and ATP; decreases ROS; increases SOD2 and catalase; involves a Ca^{2+}-dependent mechanism	Indirect mechanistic evidence	Low [169]
Rg3 monotherapy; 0.1–1 μM in vitro	C2C12 myotubes; dexamethasone-induced myotube atrophy	Mitochondrial function	Activates Akt/mTOR/p70S6K signaling; decreases Atrogin-1 and MuRF1; increases PGC-1α activity, Nrf1, and TFAM; decreases mitochondrial ROS and increases MMP and ATP	Indirect mechanistic evidence	Low to moderate [170]
Rg3 monotherapy; 0.01–1 μM in vitro	TNF-α-induced atrophy in C2C12 myotubes	Mitochondrial biogenesis	Activates Akt/mTOR/p70S6K signaling; decreases FoxO3, Atrogin-1, and MuRF1; increases PGC-1α activity, Nrf1, and TFAM; decreases mitochondrial ROS and increases MMP and ATP	Indirect mechanistic evidence	Low to moderate [171]
BST204 (Rg3 15.0% + Rh2 6.1%; mixture); 3–25 μg/mL in vitro	TNF-α-induced atrophy in C2C12 myotubes	Muscle metabolism/mitochondrial function	Activates Akt/mTOR/p70S6K signaling; decreases FoxO3, Atrogin-1, and MuRF1; increases PGC-1α activity, Nrf1, and TFAM; decreases mitochondrial ROS and increases MMP and ATP	Indirect mechanistic evidence	Low to moderate [172]
Rg1; 20/40/80 mg/kg/day, i.p., for 14 days in vivo; 12.5/25/50 μmol/L in vitro	Bone marrow nucleated cells from patients with aplastic anemia (AA; *n* = 8); an AA mouse model induced by ^{60}Co irradiation and DBA/2 lymph node cells; lineage-negative, Sca-1-positive, c-Kit-positive cells in vivo and in vitro	Hematopoiesis	Inhibits mitochondrial translocation of Bax; increases the Bcl-2/Bax ratio; decreases ROS, Cyt-C, Apaf-1, AIF, and cleaved caspase-3; increases mitochondrial number and mtDNA content; improves ATP/ADP and AMP profiles and MAO/COX activity curves	Indirect mechanistic evidence	Low to moderate [173]
Rb1; 1/2/4 mg/mL solution, 0.2 mL, i.p., once daily for 12 days; mg/kg dose not specified	Immune-mediated AA mouse model (sublethal ^{60}Co irradiation plus DBA/2 lymphocytes)	Hematopoiesis	T-cell subsets: decreases $CD3^+CD8^+$ T cells and increases $CD3^+CD4^+$ T cells and the $CD4^+/CD8^+$ ratio. Dendritic cells: inhibits DC maturation, with reduced CD86 expression at medium and high doses	Indirect mechanistic evidence	Low to moderate [174]

Compound/Dose	Experimental model	Mechanistic focus	Key Molecular Targets and Pathways	Evidence classification	Evidence level
Rg1; 10/15 mg/kg in vivo; 5/10/15 μM in vitro; treatment duration, 13 days	Cyclophosphamide (CTX)-induced bone marrow suppression/AA in C57BL/6 mice; in vitro bone marrow hematopoietic stem cells	Hematopoiesis	MAPK signaling: decreases the p-p38/p38, p-JNK/JNK, and p-ERK/ERK ratios; decreases caspase-3/9 and Bax and increases Bcl-2; decreases IL-6, TNF-α, IL-1β, and CCL2 and increases IL-10; alleviates cell cycle arrest	Indirect evidence related to cancer treatment	Moderate [175]
Rg3; 10–500 μg/mL in proliferation assays	Primary spleen and bone marrow (BM) cells from male ICR mice (5–6 weeks); cultured in vitro with or without cyclophosphamide (CYC, 1.6 μg/mL)	Hematopoiesis	Hematopoiesis-related targets: SCF, IL-3, GM-CSF, and TPO mRNA (upregulated at 36 h); promotes spleen/BM cell proliferation and hematopoietic progenitor colony formation.	Indirect mechanistic evidence	Low [176]

* The full forms of the abbreviations are provided in the list below. Ginsenosides are denoted as Rg1, Rb1, Rg3, Rh2, Rg5, Rz1, and Rk1 according to standard nomenclature; 20(R)-Rg3 denotes the 20(R) epimer.Arrows (↑ and ↓) indicate increases and decreases, respectively. The prefix "p-" denotes the phosphorylated form of the corresponding protein.

Table S2. Study Designs and Intervention Protocols of Existing Clinical Studies Investigating Ginseng for CRF.

First author, Year	Sample/Study Design	Ginseng Preparation	Control/Duration	Adverse Events
Barton et al. (2010) [183]	Randomized, double-blind, placebo-controlled, dose-finding pilot study; 290 participants enrolled, 282 randomized, and 175 completed the study.	American ginseng root capsules; approximately 5% ginsenosides; 750, 1000, or 2000 mg/day.	Placebo; 8 weeks	No significant between-group differences in toxicity.
Barton et al. (2013) [184]	Multicenter, randomized, double-blind, placebo-controlled RCT; 364 participants randomized, of whom 341 initiated treatment.	American ginseng root powder from Wisconsin; approximately 3% ginsenosides; single production batch; 2000 mg/day.	Placebo; 8 weeks	Self-reported and CTCAE-graded toxicities; no significant between-group differences.
Yennurajalingam et al. (2015) [187]	Prospective, open-label, single-arm pilot study; 30 participants received treatment, and 24 were evaluable.	Asian ginseng extract standardized to contain at least 7% ginsenosides and malonyl ginsenosides; manufacturing range of ginsenosides, 12% ± 3%.	None; 29 days	No drug-related AEs of Grade ≥3 were reported.
Yennurajalingam et al. (2017) [188]	Randomized, double-blind, placebo-controlled RCT; 127 participants randomized, of whom 112 were evaluable.	Standardized Asian ginseng extract; 400 mg twice daily (800 mg/day).	Placebo; 28 days	Any-grade toxicity: 28/63 vs. 33/64 (p = 0.024); One grade ≥3 infection/infections and infestations event occurred in the ginseng group.

First author, Year	Sample/Study Design	Ginseng Preparation	Control/Duration	Adverse Events
Pourmohamadi et al. (2018) [189]	Prospective, randomized, placebo-controlled study; blinding NR. The main text reports 114 participants (ginseng, n = 54; placebo, n = 60), whereas the abstract reports 113 participants.	Asian ginseng; 100 mg/day; specific ginsenoside content NR.	Placebo; 30 days	Specific AEs were not systematically reported; the authors reported no apparent adverse consequences.
Chang et al. (2018) [186]	Single-center retrospective chart review; 28 participants received combination therapy, and 15 completed the study. No randomization or blinding was used.	American ginseng; 2000 mg/day; methylphenidate (10–40 mg/day) co-administered in some participants; ginsenoside content NR.	No control group; mean duration of 30.5 days	Three patients had grade 2 adverse effects per the abstract; in the main text, two discontinued methylphenidate because of grade 2 anxiety/diarrhea, and one discontinued for personal reasons.
Kim et al. (2020) [185]	Multicenter, randomized, double-blind, placebo-controlled RCT; 438 participants randomized (219 per group), with 409 included in the full analysis set.	Korean red ginseng; total content of seven ginsenosides, 1.49% ± 0.4%; Rb1 content, 8.03 mg/g; 2000 mg/day.	Placebo; 16 weeks	Neutropenia was more frequent in the KRG group; overall AE incidence was similar between groups.
Guglielmo et al. (2020) [190]	Randomized, double-blind, placebo-controlled RCT; 32 participants randomized (17 vs. 15).	American ginseng (P. quinquefolius; Equunax®, SCM Health); 1000 mg/day (two 500-mg capsules daily); ginsenoside content NR.	Placebo; 8 weeks	AEs were not systematically reported in the results section; therefore, the overall acceptability of the safety profile could not be confirmed.
Wang et al. (2024) [191]	Prospective, open-label, nonrandomized, controlled multicenter cohort study; 529 participants enrolled and allocated to groups (261 vs. 268), with 409 included in the final analysis (RSGB, n = 206; control, n = 203).	Renshen Guben Oral Liquid; 10 mL twice daily (20 mL/day); specific ginsenoside content NR; multi-herb formulation.	Supportive care; 8 weeks	Overall AEs: 31.0% vs. 34.0%; Grade ≥3 AEs: 8.9% vs. 10.7%; no serious drug-related AEs were observed.

* The full forms of the abbreviations are provided in the list below. NR indicates that the corresponding information was not explicitly reported in the original article.

Table S3. Clinical Findings, Limitations, and Evidence Assessment of Ginseng for CRF.

First author, Year	Main Results	Limitations	Level of Evidence
Barton et al. (2010) [183]	The pooled ginseng groups showed no significant difference from placebo in the AUC for BFI activity interference (p = 0.21). A nonsignificant trend toward improvement was observed at 1000–2000 mg/day. At 2000 mg/day, the effect size was approximately 0.56 SD.	Heterogeneity in study populations; variability in outcome measurements; issues with preparation standardization; unclear dose–response relationship.	Level 1b (RCT/exploratory pilot study; not equivalent to a confirmatory trial)

First author, Year	Main Results	Limitations	Level of Evidence
Barton et al. (2013) [184]	Change from baseline in the MFSI-SF general subscale: 14.4 vs. 8.2 at week 4 (P = 0.07; MD = 6.2) and 20.0 vs. 10.3 at week 8 (P = 0.003; MD = 9.7). No significant differences were observed in the BFI total score or activity interference. Calculated value: MD = 6.2, MD = 9.7; 95% CI NR.	Short treatment duration (8 weeks); inconsistencies across outcome measures; dose–response relationship could not be assessed with a single dose.	Level 1b (single RCT)
Yennurajalingam et al. (2015) [187]	FACIT-F change: +14.21 ± 17.54 (p = 0.0006); ESAS fatigue score change: −2.46 ± 2.15 (p = 0.0001). By day 15, 87% of participants showed an improvement of ≥3 points. MD NR.	No control group; small sample size; potential confounding by cancer treatment; exploratory efficacy findings; dose–response relationship could not be assessed.	Level 4 (single-arm, open-label pilot study; low certainty of evidence)
Yennurajalingam et al. (2017) [188]	FACIT-F change scores: 7.5 ± 12.7 vs 6.5 ± 9.9 (p = 0.67); not statistically significant. Clinical improvement rates were 55.5% vs. 54.6%. 95% CI NR and calculated value: MD was approximately 1.0.	Short treatment duration; single dose; long-term efficacy unknown.	Level 1b (single RCT; primary endpoint not statistically significant)
Pourmohamadi et al. (2018) [189]	The proportion classified as “good” on the BEK/Beck assessment increased from 24.1% to 81.5%, while the proportion with moderate depression decreased from 55.6% to 18.5% (p < 0.0001). The placebo group showed no significant change (p = 0.887). The scale was not standardized, MD and 95% CI NR.	Small sample size; limited description of randomization; unclear blinding procedures; non-standardized outcome measures; short treatment duration; single dose.	Level 1b (single RCT; methodological limitations and low certainty of evidence)
Chang et al. (2018) [186]	ESAS fatigue scores decreased from 6.93 to 4.13 (change: −2.8 ± 1.61; p = 0.0002). A reduction of ≥3 points was observed in 60% of participants, MD NR.	Retrospective design; small sample size; no control group; concomitant methylphenidate use precludes attribution of effects to ginseng alone; single dose.	Level 4 (retrospective case series; very low certainty of evidence)
Kim et al. (2020) [185]	In the full analysis set, the between-group difference in global BFI AUC was 2.41 (p = 0.119). Significant differences were observed for the “mood” (p = 0.038; difference = 3.49) and “walking ability” (p = 0.023; difference = 4.03) domains. In the per-protocol (PP) set, the global BFI result was significant (p = 0.019); 95% CI NR.	Inconsistency between full analysis set and per-protocol results; single dose, precluding dose–response assessment; limited information on preparation standardization.	Level 1b (single RCT; primary endpoint not statistically significant in the full analysis set; results should be interpreted cautiously)
Guglielmo et al. (2020) [190]	At week 8, BFI scores were 4.59 vs. 3.44, with no statistically significant between-group difference. Changes from baseline were 1.83 vs. 2.41, also with no significant difference. One activity-interference item favored placebo (p = 0.018). The between-group difference in change scores was MD = 0.58; 95% CI NR.	Small sample size and limited statistical power; short treatment duration; single dose, precluding dose–response assessment.	Level 1b (single RCT/Phase II trial; low certainty of evidence)
Wang et al. (2024) [191]	No significant improvements were observed at week 4. At week 8, significant improvements were reported in MFI, BFI, and FACT-Hep scores (p < 0.001, p < 0.001, and p = 0.002, respectively). In Model 1, the estimated effects were as follows: MFI, −20.20 (95% CI: −26.08 to −14.32); BFI, −1.73 (95% CI: −2.26 to −1.20); and FACT-Hep, −9.60 (95% CI: −15.52 to −3.67).	Nonrandomized, open-label design; potential confounding; short treatment duration; single dose; multi-herb formulation limits comparability with single-herb ginseng preparations.	Level 2b (prospective controlled cohort study; nonrandomized and open-label; limited certainty of evidence)

* The full forms of the abbreviations are provided in the list below. NR, data not explicitly reported in the original article. Evidence levels were assigned according to study design using the 2011 criteria of the Oxford Centre for Evidence-Based Medicine (CEBM). When evidence levels were not reported in the original articles, they were assigned based on study design. Evidence levels reflect the methodological hierarchy of study designs and should not be interpreted as equivalent to the overall certainty of evidence or the reliability of treatment effects.

Abbreviations

The abbreviations used in Supplementary Tables S1–S3 are defined below. They are grouped by category to improve readability and are presented in semicolon-separated prose rather than in tabular form. General statistical, formatting, and evidence-level notations such as *n*, *p*, vs., et al., Grade ⩾3, and Level 1b/4 are not included.

Disease models, study designs, samples, and assessment instruments. AA, aplastic anemia; AE/AEs, adverse event(s); AUC, area under the curve; BFI, Brief Fatigue Inventory; CEBM, Oxford Centre for Evidence-Based Medicine; CI, confidence interval; CRF, cancer-related fatigue; CTCAE, Common Terminology Criteria for Adverse Events; CTD, cantharidin; CUMS, chronic unpredictable mild stress; EEG, electroencephalogram; EMG, electromyogram; ESAS, Edmonton Symptom Assessment System; 5-FU, 5-fluorouracil; FACIT-F, Functional Assessment of Chronic Illness Therapy–Fatigue; FACT-Hep, Functional Assessment of Cancer Therapy–Hepatobiliary; FST, forced swimming test; GDX, gonadectomy; HPA, hypothalamic–pituitary – adrenal axis; KRG, Korean red ginseng; LPS, lipopolysaccharide; MD, mean difference; MFI, Multidimensional Fatigue Inventory; MFSI-SF, Multidimensional Fatigue Symptom Inventory–Short Form; NR, not reported; BEK/Beck, Beck assessment (instrument name as reported in the original study); PFC, prefrontal cortex; POFS, postoperative fatigue syndrome; PP, per-protocol (per-protocol analysis set); RCT, randomized controlled trial; RSGB, Renshen Guben Oral Liquid; SD, standard deviation; TBI, traumatic brain injury; TST, tail suspension test; WEG/GE5/GE50, ginseng extract comparator preparations (WEG：Water Extract of Ginseng); C26, Colon 26 carcinoma cell line; RAW264.7, mouse macrophage cell line RAW264.7; THP-1, human monocytic leukemia cell line THP-1; SH-SY5Y, human neuroblastoma cell line SH-SY5Y; C57BL/6, C57 black 6 mouse strain; C57BL/6J, C57BL/6 Jackson substrain; DBA/2, DBA/2 mouse strain; ICR, Institute of Cancer Research mouse strain; ^{60}Co, cobalt-60; p.o., per os (oral administration); i.p., intraperitoneal injection; i.g., intragastric administration; CTX, cyclophosphamide; CYC, cyclophosphamide.

Molecular mediators, signaling pathways, and immune markers. ac-p65, acetylated NF-κB RelA subunit; AR, androgen receptor; TNF-α, tumor necrosis factor-α; IL-1β, interleukin-1β; IL-3, interleukin-3; IL-6, interleukin-6; IL-10, interleukin-10; TLR4, Toll-like receptor 4; NF-κB, nuclear factor kappa-B; NLRP3, NOD-like receptor family pyrin domain containing 3; TNBS, 2,4,6-trinitrobenzene sulfonic acid; IRAK1, interleukin-1 receptor-associated kinase 1; TAK1, transforming growth factor-β-activated kinase 1; IKKα/β, inhibitor of nuclear factor κB kinase α/β; IκBα, inhibitor of nuclear factor κB α; p65, NF-κB RelA subunit; Th17, T helper 17 cells; Treg, regulatory T cells; RORγt, retinoic acid receptor-related orphan receptor gamma t; miR-21, microRNA-21; NMIIA, non-muscle myosin IIA; HSP90, heat shock protein 90; TIMP3, tissue inhibitor of metalloproteinases 3; MMP-1/3/9, matrix metalloproteinases 1/3/9 (tissue remodeling/blood–brain barrier context); ZO-1, zonula occludens-1; Iba1, ionized calcium-

binding adapter molecule 1; iNOS, inducible nitric oxide synthase; COX-2, cyclooxygenase-2 (inflammation context); SIRT1, sirtuin 1; MAPK, mitogen-activated protein kinase; JNK1/2, c-Jun N-terminal kinase 1/2; p38, p38 MAPK; ERK1/2, extracellular signal-regulated kinase 1/2; MD2, myeloid differentiation factor 2; MyD88, myeloid differentiation primary response 88; Ca^{2+}, calcium ion; ROS, reactive oxygen species; NO, nitric oxide; PI3K, phosphoinositide 3-kinase; Akt, protein kinase B; mTOR, mammalian target of rapamycin; FoxO, forkhead box protein O (includes FoxO1, FoxO3a isoforms); Nrf2, nuclear factor erythroid 2-related factor 2; p85, PI3K regulatory subunit p85; MDA, malondialdehyde; 8-iso-PGF2α, 8-iso-prostaglandin F2α; SOD, superoxide dismutase; SOD2, superoxide dismutase 2 (manganese superoxide dismutase); RNA, ribonucleic acid; DNA, deoxyribonucleic acid; mRNA, messenger RNA; CORT/Cort, corticosterone; CRH, corticotropin-releasing hormone; ACTH, adrenocorticotropic hormone; HPG axis, hypothalamic – pituitary – gonadal axis; PVH, paraventricular hypothalamus; LH, lateral hypothalamus; c-Fos, Fos proto-oncogene protein; 5-HT, 5-hydroxytryptamine/serotonin; 5-HIAA, 5-hydroxyindoleacetic acid; GABA, γ-aminobutyric acid; Glu, glutamate; NE, noradrenaline; ACh, acetylcholine; BDNF, brain-derived neurotrophic factor; TrkB, tropomyosin receptor kinase B; Bcl-2, B-cell lymphoma 2; Bax, Bcl-2-associated X protein; DA, dopamine; 5-HTP, 5-hydroxytryptophan; DOPA, dihydroxyphenylalanine; EP, epinephrine; DOPAC, 3,4-dihydroxyphenylacetic acid; HVA, homovanillic acid; TH, tyrosine hydroxylase; PKAα, protein kinase A alpha catalytic subunit; Ser40, serine 40; IDO, indoleamine 2,3-dioxygenase; Ym-1, chitinase 3-like 3; PGE_2, prostaglandin E_2; MerTK, MER proto-oncogene tyrosine kinase; MuRF1, muscle RING-finger protein 1; NADH-TR, NADH tetrazolium reductase; SDH, succinate dehydrogenase; SDHB, succinate dehydrogenase complex iron-sulfur subunit B; OXPHOS, oxidative phosphorylation; COX, cytochrome c oxidase (mitochondrial respiration context); COX2, cytochrome c oxidase subunit 2; MB, myoglobin; GLUT4, glucose transporter type 4; HK, hexokinase; PK, pyruvate kinase; PFK, phosphofructokinase; S6K, ribosomal protein S6 kinase (p70S6K, 70 kDa isoform); 4EBP1, eukaryotic translation initiation factor 4E-binding protein 1; MHC I/IIA/IIB/IIX, myosin heavy chain isoforms I/IIA/IIB/IIX (not major histocompatibility complex); CSA, cross-sectional area; PGC-1α, peroxisome proliferator-activated receptor gamma coactivator 1-alpha; NRF1, nuclear respiratory factor 1; TFAM, mitochondrial transcription factor A; TFEB, transcription factor EB; Tom20, translocase of outer mitochondrial membrane 20; UQCRC2, ubiquinol-cytochrome c reductase core protein 2; MMP, mitochondrial membrane potential (mitochondrial function context; note distinct from matrix metalloproteinases MMP-1/3/9); OCR, oxygen consumption rate; ATP, adenosine triphosphate; ADP, adenosine diphosphate; AMP, adenosine monophosphate; Cyt-C, cytochrome c; Apaf-1, apoptotic protease-activating factor 1; AIF, apoptosis-inducing factor; mtDNA, mitochondrial DNA; MAO, monoamine oxidase; BM, bone marrow; SCF, stem cell factor; GM-CSF, granulocyte–macrophage colony-stimulating factor; TPO, thrombopoietin; Sca-1, stem cell antigen-1; c-Kit, tyrosine-protein kinase Kit/CD117; CD3, cluster of differentiation 3; CD4, cluster of differentiation 4; CD8, cluster of differentiation 8; CD86, cluster of differentiation 86; DC, dendritic cell; CCL2, C-C motif chemokine ligand 2; GR, glucocorticoid receptor; IL-12p40, interleukin-12 p40 subunit; IL-17A, interleukin-17A; IL-21, interleukin-21; IL-22, interleukin-22; MPO, myeloperoxidase; Na^+/K^+-ATPase, sodium/potassium-transporting ATPase; SIRT3, sirtuin 3; TGF-β, transforming growth factor beta.
